# A novel strain of cynomolgus macaque cytomegalovirus: implications for host-virus co-evolution

**DOI:** 10.1186/s12864-016-2588-3

**Published:** 2016-04-05

**Authors:** Justen N. Hoffman Russell, Angie K. Marsh, David O. Willer, Aruna P. N. Ambagala, Misko Dzamba, Jacqueline K. Chan, Richard Pilon, Jocelyn Fournier, Michael Brudno, Joseph M. Antony, Paul Sandstrom, Ben J. Evans, Kelly S. MacDonald

**Affiliations:** Department of Immunology, University of Toronto, Toronto, M5S 1A8 ON Canada; Department of Medicine, University of Toronto, Toronto, M5S 1A8 ON Canada; Department of Microbiology, Mount Sinai Hospital, Toronto, M5G 1X5 ON Canada; Department of Computer Science, University of Toronto, Toronto, M5S 1A8 ON Canada; National HIV & Retrovirology Laboratories, Public Health Agency of Canada, Ottawa, K1A 0K9 ON Canada; Scientific Services Division, Health Products & Food Branch, Health Canada, Ottawa, K1A 0L2 ON Canada; Biology Department, McMaster University, Life Sciences Building, 1280 Main Street West, Hamilton, L8S 4K1 ON Canada; Section of Infectious Diseases, Department of Internal Medicine, University of Manitoba, 745 Bannatyne Ave, Winnipeg, R3E 0J9 MB Canada; Present Address: Canadian Science Centre for Human and Animal Health, National Centre for Foreign Animal Disease, 1015 Arlington Street, Winnipeg, R3E 3M4 MB Canada; Present Address: University of Manitoba, Basic Medical Sciences Building, Room 501, 745 Bannatyne Ave., Winnipeg, R3E 0J9 MB Canada

**Keywords:** Cynomolgus macaque, Cytomegalovirus, Phylogenetics, Co-speciation, Co-evolution

## Abstract

**Background:**

Cytomegaloviruses belong to a large, ancient, genus of DNA viruses comprised of a wide array of species-specific strains that occur in diverse array of hosts.

**Methods:**

In this study we sequenced the ~217 Kb genome of a cytomegalovirus isolated from a Mauritius cynomolgus macaque, CyCMV Mauritius, and compared it to previously sequenced cytomegaloviruses from a cynomolgus macaque of Filipino origin (CyCMV Ottawa) and two from Indian rhesus macaques (RhCMV 180.92 and RhCMV 68–1).

**Results:**

Though more closely related to CyCMV Ottawa, CyCMV Mauritius is less genetically distant from both RhCMV strains than is CyCMV Ottawa. Several individual genes, including homologues of CMV genes *RL11B, UL123*, *UL83b*, *UL84* and a homologue of mammalian COX-2, show a closer relationship between homologues of CyCMV Mauritius and the RhCMVs than between homologues of CyCMV Mauritius and CyCMV Ottawa. A broader phylogenetic analysis of 12 CMV strains from eight species recovers evolutionary relationships among viral strains that mirror those amongst the host species, further demonstrating co-evolution of host and virus.

**Conclusions:**

Phylogenetic analyses of rhesus and cynomolgus macaque CMV genome sequences demonstrate co-speciation of the virus and host.

**Electronic supplementary material:**

The online version of this article (doi:10.1186/s12864-016-2588-3) contains supplementary material, which is available to authorized users.

## Background

Macaque monkeys are an important animal model in biomedical research, particularly in infectious diseases. They are used in vaccine and infectivity studies of viruses that either do not infect or do not produce realistic pathogenic features in small animal models – viruses such as Human Immunodeficiency Virus (HIV) where Simian Immunodeficiency Virus (SIV) or the human envelope substituted version (SHIV) can be studied as a surrogate in macaques.

Recently, the limited availability of Indian rhesus macaques in North America and elsewhere has led to the development of resources and standards for the use of cynomolgus macaques and Chinese rhesus macaques [1], the former in particular being an excellent model of HIV-1/SIV infection [2]. Though relatively closely related, divergence between populations or species of macaques and their corresponding cytomegaloviruses (CMV) strains is substantial enough as to preclude direct replacement in any study. In fact, with increased usage in research, it has been questioned whether cynomolgus macaques may be in need of taxonomic re-classification [3]. Captive populations of cynomolgus macaques are frequently interbred with little concern as to their origins. However, the genetic divergence between geographically distinct populations of cynomolgus macaques rivals that found between Indian and Chinese rhesus macaques, a factor that has the potential to differently influence experimental results [3]. For example, disease susceptibility is highly variable between isolated-tightly knit groups of macaques found across the Sunda Shelf of Southeast Asia. Macaque genome sequencing has enabled a better understanding of their disease susceptibility from an evolutionary and conservation standpoint, and provided insight into population structure and patterns of migration [3–6].

Interest in nonhuman primate herpesviruses, and CMVs in particular, has increased in recent years in recognition of the unique immune response that they evoke. Specifically, CMV evokes a type of effector memory T cell response, which intermittently is boosted by reactivated virus throughout the life of its immunocompetent host. This type of immune memory qualitatively is different from conventional T cell memory in that it does not require priming and can respond immediately to antigen. Thus CMVs are also of interest to vaccinologists who are examining ways to expand the duration of rapidly inducible T cell responses to block primary viral infection. The evaluation of CMVs in rhesus and more recently, in cynomolgus macaques, has become a priority in order to facilitate the use of these animal models for vaccine research and development. CMV infects many primate and non-primate hosts including humans, baboons, green monkeys, chimpanzees, squirrel monkeys, macaques, oysters and rodents [7]. Since previous studies have demonstrated only limited mixing and horizontal transmission between mammalian populations [8], it is thought that CMV, like other herpesviruses, diversified via co-evolution with their host species [8, 9].

During speciation, pathogens may co-speciation with their host [10]. Herpesviruses, for instance, co-evolve with their mammalian hosts [11]. Herpesviruses were present in primates 70 mya [12], and have been used as surrogates to track mammalian – including human – evolution and migration [12, 13]. Herpesviruses, including CMVs, were also present in the most recent common ancestor of rhesus and cynomolgus macaques, and are presumed to have undergone geographic differentiation similar to their host species [14]. However, macaques can become infected with multiple strains of CMV and these CMV strains may experience recombination. Hosts co-infected with two CMV strains occasionally release infectious viral particles (shed virus) from multiple strains (dual shedding), but generally have a dominant strain that makes up the majority of shed virus [15]. Interaction among populations of cynomolgus macaques can facilitate the exchange of CMV strains [16] but cross infection between species is generally rare [17].

In the present study, we sequenced a new strain of CyCMV from a cynomolgus macaque from Mauritius and compared it to three other strains – one from a cynomolgus macaque that originated in the Philippines (CyCMV Ottawa) [18], and two from Indian rhesus macaques (RhCMV), RhCMV 68–1 [19] and RhCMV 180.92 [20]. We also characterized variation in gene content among these macaque CMVs. Our results indicate that evolutionary relationships over the complete genomes of cynomolgus CMV strains matches those of their hosts, thereby supporting their co-evolution and also the further use of CMVs in the study of mammalian biogeography and phylogeny. We also found phylogenetic correspondence between CMVs and their hosts in a broader phylogenetic analysis that included several other CMV strains. However, phylogenetic relationships among some genomic regions of the macaque CMVs deviated from the expected relationship, and this can be explained either by intra-strain recombination or alternatively could reflect phylogenetic error.

## Results and discussion

### CyCMV Mauritius is a unique CMV from Mauritian cynomolgus macaques

In the present study, we characterized a novel CMV strain, isolated from a Mauritian cynomolgus macaque, and compared its sequence to that of three other CMV strains, including that of CyCMV Ottawa [18], and two strains from rhesus macaques: RhCMV 68–1 [19] and RhCMV 180.92 [20]. Our analyses indicate that the sequence of CyCMV Mauritius is highly similar to the sequence of CyCMV Ottawa, and also that both are derived from a more recent common ancestor than either is with one of the rhesus macaque strains.

Our assembly of Illumina reads from CyCMV Mauritius produced three contigs that were then connected by Sanger sequencing. The assembly had an average 280X fold coverage that was 217 200 bp in length, and had 49.5 % GC content. In comparison, CyCMV Ottawa is 841 bp longer and has a similar GC content (Table 1). RhCMV 68–1 and RhCMV180.92 are both similar in length and GC content, with RhCMV 180.92 being the shortest and RhCMV 68–1 the longest of the four viruses. Compared to other mammalian CMVs, all macaque CMVs examined are shorter than chimpanzee CMV (CCMV) Heberling strain and shorter on average than Human CMV (HCMV) strains AD169 and HAN1. Of the CMVs that have been sequenced thus far, these four macaque CMVs are most similar in length to Aotine (Owl Monkey) CMV and African Green Monkey CMV.

**Table 1:** Select sequenced primate CMV strains

Virus	Strain	Genbank Asscession	Host Species	Length (bp)	%GC
CyCMV	Mauritius	KP796148	Cynomolgus Macaque	217 200	49.5 %
CyCMV	Ottawa	JN227533	Cynomolgus Macaque	218 041	49.5 %
RhCMV	180.92	DQ120516	Rhesus Macaque	215 678	49.0 %
RhCMV	68-1	AY186194	Rhesus Macaque	221 454	49.0 %
OMCMV	S34E	FJ483970	Owl Monkey	219 474	56.3 %
GMCMV	Colburn	FJ483969	African Green Monkey	219 526	51.2 %
GMCMV	2715	FJ483968	African Green Monkey	226 205	50.8 %
CCMV	Heberling	AF480884	Chimpanzee	241 087	61.7 %
HCMV	AD169	FJ527563	Human	229 354	57.2 %
HCMV	HAN1	JX512199	Human	235 006	62.4 %

CyCMV Mauritius and CyCMV Ottawa share 95.3 % identity by linear full genome alignment while RhCMV 68–1 and RhCMV 180.92 share 95.6 % identity. CyCMV Mauritius and RhCMV 180.92 have 87.9 % identity and CyCMV Mauritius and RhCMV 68–1 have 89.7 % identity. CyCMV Ottawa has 89.8 % identity with RhCMV 68–1 and 88.2 % identity with RhCMV 180.92.

**Fig. 1:**
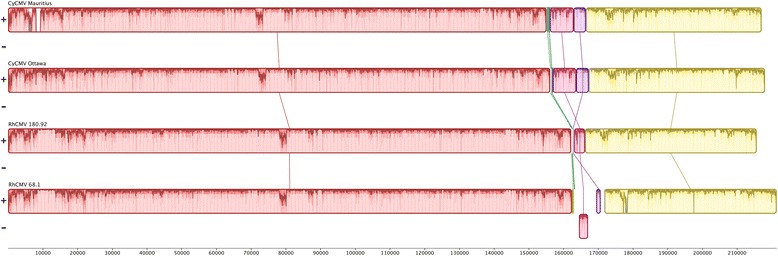
Multiple genome alignment of CyCMV Mauritius, CyCMV Ottawa, RhCMV 68–1, and RhCMV 180.92. Genome homology and rearrangement are presented using the MAUVE multiple genome alignment algorithm. Locally collinear blocks are indicated differentiated by colour and connected by linking line. Within collinear blocks column height indicates average conservation of base pairs locally between homologous collinear blocks in other strains, full columns indicate regions fully conserved between species while empty columns indicate unique regions to the particular genome or absences. Collinear blocks shown above the x-axis are in the same orientation, with those shown below reversed in comparison to the same co-linear blocks in other species

Due to the possibility of rearrangements in these viral genomes we evaluated sequence synteny using the progressive Mauve multiple genome alignment algorithm [21]. This analysis reveals a local co-linear region around 160–170 kbp where the RhCMV 68–1 sequence is reversed in comparison to CyCMV Mauritius, CyCMV Ottawa and RhCMV 180.92 (Fig. 1). This region is immediately adjacent to the *UL128* to *UL130* deletion found in RhCMV 68–1. There are two locally collinear blocks whose order are reversed between rhesus (RhCMV 68–1 and RhCMV 180.92) and cynomolgus (CyCMV Mauritius and CyCMV Ottawa) macaque CMV genomes. Both collinear blocks are short and located immediately upstream of the reversed region in RhCMV 68–1.

### Phylogenetic relationships among CMV strains match those of their hosts

**Fig. 2:**
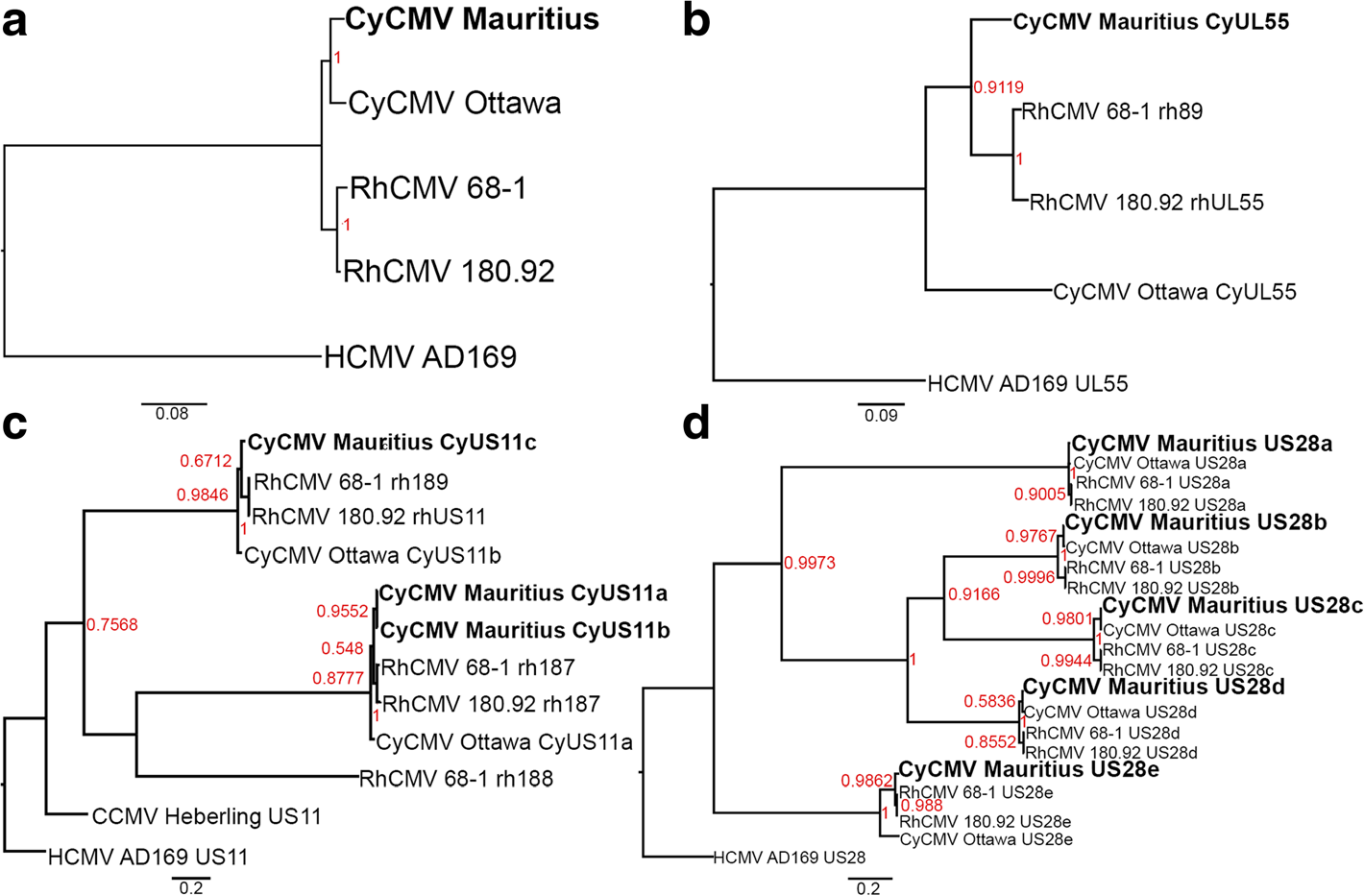
Phylogenetic tree comparison of Macaque CMV genome and select genes. Trees were generated comparing CyCMV Mauritius, CyCMV Ottawa, RhCMV 68–1 and RhCMV 180.92 using HCMV AD169 as an out-group. Trees were generated using Mr. Bayes, following MAFFT alignment, using a model of evolution selected by JModel test **a**) from a full genome global alignment; **b**) from an alignment of putative gB (UL55) ORF, a typical marker of phylogeny; **c**) from an alignment of US11, an MHC-I down-regulatory gene necessary for superinfection of CMV, Chimpanzee CMV is included; **d**) from an alignment of US28 genes, for which 5 copies are encoded in each macaque CMV (4 copies of the gene and one copy of a nonfunctional pseudogene for RhCMV 68–1). CyCMV genes CyUS28a-CyUS28e and RhCMV genes rh214-rh220 are relabeled as US28a – US28e, inclusive of the RhCMV 68–1 pseudogene homologous to other macaque CMV US28c, based on order in genome. Numbers at loci indicate posterior probability with color scaled according to probability; genetic distances measured in substitutions per site (sps) are given by scale below

Evolutionary relationships were estimated for CyCMV Mauritius, CyCMV Ottawa, RhCMV 180.92 and RhCMV 68–1 using HCMV AD169 as an outgroup (Fig. 2). Evolutionary relationships among these complete macaque CMV genome sequences match those of their hosts (Fig. 2a). Within the cynomolgus macaque, the viral strains CyCMV Mauritius and CyCMV Ottawa are diverged by 0.036 substitutions per site (sps), whereas within the rhesus macaque, the viral strains RhCMV 180.92 and RhCMV 68–1 are diverged by only 0.017 sps. Thus, divergence between CMV strains of cynomologous macaques is greater than between the CMV strains of rhesus macaques, and this may also be true of the respective host populations.

All four-macaque CMVs share similar genome architecture. Macaque CMV and the CMV of closely related primates, like green monkey CMVs (GMCMVs) Colburn and 2715, have terminal repeats, as well as unique long and unique short regions in genome structure, but lack the internal repeat regions of other primate CMVs [7].

### CyCMV genomes show diversity in gene conservation

**Fig. 3:**
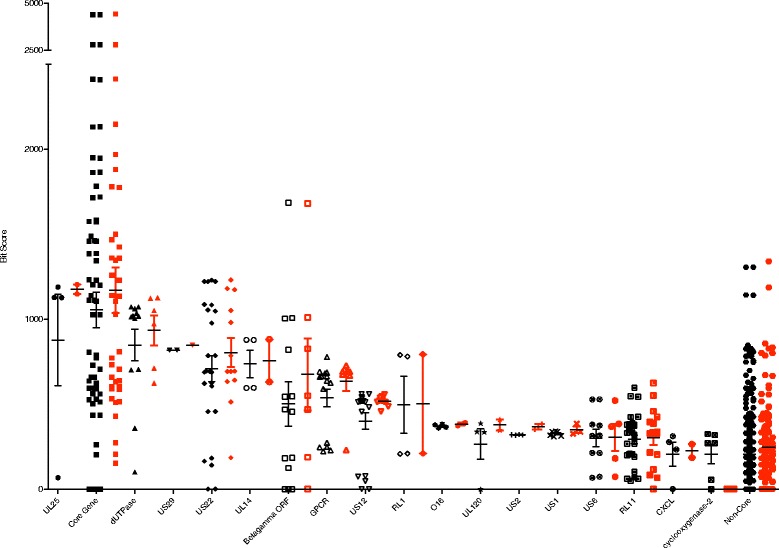
Conservation of ORFs of known gene families. Bit-scores are plotted for ORFs of CyCMV Mauritius to homologous ORFs of RhCMV 68–1 and RhCMV 180.92 (*Black*) and to homologous ORFs of CyCMV Ottawa (*Red*) grouped by gene gamily. Each point represents a single bit-score comparison for a single ORF. Horizontal line indicates the mean ± standard deviation

Viral genes that are important for host specificity may tend to be more diverged among strains in different hosts. To explore these possibilities, we compared divergence and gene content of the four completely sequenced macaque CMV strains in order to better understand how these factors varied among strains. Annotation of the sequenced CyCMV Mauritius genome identified 290 putative open reading frames (ORFs, Additional file 1: Figure S1; Additional file 2: Table S1). By contrast, CyCMV Ottawa has 262 putative ORFs [18], RhCMV 68–1 has 230 ORFs [19], and RhCMV 180.92 has 258 ORFs [20]. To better understand the conservation of individual ORFs in these CMV genomes we used bit-score, a log-scaled measure indicating the size of a random search string required to find an equivalently or more similar sequence than the observed match. Bit-scores were generated by comparing ORFs in each of the six pairwise comparisons between the four macaque CMV genomes. Bit-scores comparing homologous ORFs of CyCMV Mauritius and CyCMV Ottawa, RhCMV 68–1 or RhCMV 180.92 were examined grouped by gene family (Fig. 3). The average bit-score for genes in each family, but not the bit-score of each individual gene, is higher for the comparison of genes between the two CyCMVs than between CyCMV Mauritius and RhCMV 180.92 or RhCMV 68–1. The only exception to this is the COX-2 family, which is absent from CyCMV Ottawa.

**Fig. 4:**
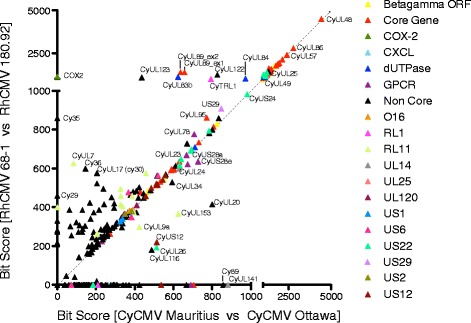
Two-dimensional bit-score plots between CMV strains. Graph represents comparisons of gene homologue bit-scores across CyCMV Mauritius versus CyCMV Ottawa and RhCMV 68–1 versus RhCMV 180.92. Bit-score among RhCMV homologues of the indicated gene are tracked on the y-axis while CyCMV homologues are tracked on the x-axis. A high bit-score indicated a high level of similarity between genes, while a bit-score of zero indicates one or both species in the comparison lacked a copy of the gene. Genes that fall on the diagonal are equally conserved in all comparisons, for example *CyUL48* (*rh78*) and *CyUL74* (*rh74*); genes that fall to one side of the diagonal are better conserved between viruses of one species than between viruses of the other species, for example COX-2 (*rh10*) which is highly conserved between RhCMV 180.92 and RhCMV 68–1, but absent from CyCMV Ottawa, and *CyUL94* (*rh129*). Genes are coloured according to known gene family. Select genes are annotated according to their CyCMV Mauritius names. Where genes belong to multiple families only one is indicated, for breakdown of chart by gene family see Additional file 8: Figure S7 or Additional file 9: Figure S8, Additional file 10: Figure S9 and Additional file 11: Figure S10 for alternative pairwise comparisons

Two-dimensional bit-score plots were used to further visualize variation in individual gene conservation in the pairwise comparisons between the four macaque CMV strains (Fig. 4), as in [18]. When plotted this way, ORFs found along the *x* = *y* diagonal have equivalent sequence conservation in both pairwise comparisons. Genes that are equally well conserved between the two RhCMVs and between the two CyCMVs (Bit Score >1000 in one comparison) cluster along the diagonal. However, this analysis of pairwise comparisons highlights a non-uniform level of sequence conservation in some genes. The cyclooxygenase-2 gene (*CyCOX2*), for example, is absent in CyCMV Ottawa. Similarly *CyTRL1,* an epithelial cell tropism factor [22], is more highly conserved between the RhCMVs than between the two CyCMVs. There was a larger discordance between the bit-scores generated comparing CyCMV genes and those comparing RhCMV for genes with a lower maximum bit-score. Of those genes with known functions, those involved in immune modulation or as temperance factors, and membrane proteins are more dispersed than other groups with many individual genes being more highly conserved in viruses from one species of macaque than the other. This could suggest that these viral genes face differing evolutionary pressures in the two macaque species.

**Fig. 5:**
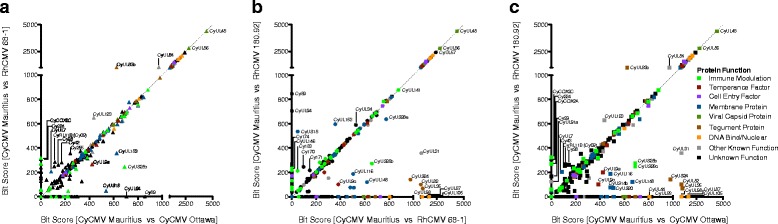
Two-dimensional bit-score plots between CMV strains. Graphs represent comparisons of gene homologue bit-scores across three strains simultaneously. Comparison of CyCMV Mauritius versus CyCMV Ottawa and CyCMV Mauritius versus RhCMV 68–1 (**a**), CyCMV Mauritius versus RhCMV 180.92 and CyCMV Mauritius versus RhCMV 68–1 (**b**), and CyCMV Mauritius versus RhCMV 180.92 and CyCMV Mauritius versus CyCMV Ottawa (**c**). Genes are coloured according to known function. Immune ORFs are involved in host immune regulation or evasion, entry ORFs are known cell entry factors, temperance ORFs are involved in temperance of viral growth, membrane ORFs are presented in cell membrane on infected cells or in virions, DNA/nuclear are known to interact with DNA or shuttle to the cell nucleus, capsid ORFs form the virus capsid, tegument proteins are found in the viral tegument, the function of ORFs with other known function vary, and the function is unknown at the time of writing for ORFs plotted as unknown. Individual ORF may have several functions; see Additional file 3: Figure S2, Additional file 4: Figure S3, Additional file 5: Figure S4 and Additional file 6: Figure S5 for visualization without overlap. Select genes are annotated according to their CyCMV Mauritius names

When grouped by gene function, capsid, DNA binding/nuclear, and tegument groupings contain highly conserved genes and less deviation in bit-score from the diagonal is observed (see Fig. 5; Additional file 3: Figure S2, Additional file 4: Figure S3, Additional file 5: Figure S4, and Additional file 6: Figure S5). In each of these functional categories, genes deviate towards a higher bit-score in CyCMV Mauritius versus CyCMV Ottawa and CyCMV Mauritius versus RhCMV 68–1, and towards a lower bit-score in CyCMV Mauritius versus RhCMV 180.92. A similar trend for higher bit-scores in CyCMV Mauritius versus CyCMV Ottawa and CyCMV Mauritius versus RhCMV 68–1, and a lower bit-score in CyCMV Mauritius versus RhCMV 180.92 was observed with ORFs involved in viral temperance, though the average bit-score was lower, and was observed to a lesser absolute extent with immune regulatory, entry, and membrane associating ORF groupings.

Though closely related to CyCMV Ottawa, CyCMV Mauritius is less genetically diverged from both RhCMVs than CyCMV Ottawa is at several individual genes, including homologues of CMV genes *RL11B*, *UL123*, *UL83b*, *UL84* and a homologue of mammalian COX-2 (Additional file 2: Table S1). This suggests a non-uniform rate of evolution of some genes (Fig. 2a) and could reflect differences in selective pressure on the CMV strains in different hosts. The high average bit-score of capsid, DNA binding/nuclear, and tegument ORFs indicate a slow rate of evolution of these genes. Immune regulatory, viral entry, and membrane associated ORFs, in contrast, exhibit signs of more rapid evolution. This may be because the proteins these genes encode interact directly with the host immune system and may consequentially face greater directional or diversifying selection.

Macaque CMV genomes lack an internal repeat region between the unique long (UL) and unique short (US) regions. We explored the effect of this feature of genome architecture on the genomic location of genes using gene bit-scores. Our analyses indicate that there is more divergence between genes from different macaque CMV strains that are closer to the terminal regions of both the UL and US genome regions than between genes in different strains that are far from these genomic features (Additional file 7: Figure S6). This decrease in gene conservation near the UL/US border was observed even in the absence of an internal terminal repeat region between the UL and US regions. Additionally, these terminal areas contain a higher proportion of genes that are strain specific. The center of the UL region contains a region with a high density of highly conserved genes. This is expected since in general, the rate of evolution of genes in a virus is slower in key genes, faster in less important genes, and fastest in non-coding and non-regulatory regions [23, 24], and because the UL region includes a cluster of core genes that are conserved across all herpesviruses.

During CMV replication, the genome circularizes and large genome segments may be reordered, reversed or lost [25]. This mechanism could explain why the *UL128-UL131* region is absent in RhCMV 68–1, but present in RhCMV 180.92 [26], and why *rh12-rh16* is absent from CyCMV Mauritius and CyCMV Ottawa but present in both RhCMVs. Loss of the *UL128-UL131* region is typical of attenuation (that is, the a decrease in virulence after passaging) in fibroblast cell lines, and demonstrates an inability to infect endothelial and epithelial cell lines. Thus our findings do not necessarily prove an absence of this region in the original wild-type RhCMV 68–1 [7, 19, 27]. How reversal and reordering of CMV genome segments [28] affect the evolution of CMV in wild macaque populations is yet to be fully understood.

### Gene content of CyCMV Mauritius is distinguished from other strains

Several CMV genes found in CyCMV Ottawa, RhCMV 68–1 and RhCMV 180.92 are absent in CyCMV Mauritius. In particular, nine CyCMV Ottawa ORFs lack homologues in CyCMV Mauritius whereas 20 CyCMV Mauritius ORFs lack homologues in CyCMV Ottawa, and of the latter, nine have homologues that are present in both RhCMV strains (Additional file 2: Table S1). In all, 20 genes of RhCMV 68–1 and RhCMV 180.92 are not identified in CyCMV Mauritius (Table 2). Similarly, comparison to human CMV data, identified 84 ORFs present in strains of HCMV but absent in CyCMV Mauritius (Table 3). The majority of genes absent in all macaque CMV strains but present in HCMV strains have unknown functions though some, such as UL65 and UL108, are known to have effects on CMV growth kinetics (Table 3).

**Table 2:** RhCMV genes absent from CyCMV Mauritius

Gene	Alternative Gene Name	Human Homologue	Necessary for growth in Towne^a^	Rh-Human Similarity (%)^b^	Function^c^
Rh9	-	-	-	-	
Rh12	RL11F	-	-	-	
Rh13	RL11G	-	-	-	
Rh13.1	-	-	-	-	
Rh14	-	-	-	-	Membrane protein
Rh15	-	-	-	-	
Rh16	-	-	-	-	
Rh17	RL11H	UL11	-	-	early glycoprotein
Rh18	-	-	-	-	
Rh19	RL11I	UL07	-	34 %	Membrane protein
Rh38	-	-	-	-	
Rh45	-	-	-	-	
Rh77	-	-	-	-	
Rh94	-	-	-	-	
Rh96	-	-	-	-	
Rh121	-	-		-	
Rh129	RhUL94	UL94	essential	64 %	Virion Protein
Rh142.2	-	-	-	-	
Rh151.1	-	-	-	-	
Rh158	RhUL147	UL147	dispensable	-	Viral CXC Chemokine homologue

Footnotes:

^a^Based on study of gene mutation in HCMV Towne [51]

^b^Similarity annotation based on comparisons of RhCMV 68.1 and HCMV in [19]

^c^Function annotated based on studies of HCMV [52]

**Table 3:** Human genes absent from CyCMV Mauritius

Gene	CyCMV Ottawa/RhCMV 68.1 Homologue	Function^a^	Effect of deletion on viral growth kinetics^b^	Family
RL2	−/−	-	no effect	
RL3	−/−	-	no data	
RL4	−/−	-	no effect	
RL5	−/−	-	no data	RL11
RL6	−/−	-	no effect	RL11
RL7	−/−	-	no data	
RL8	−/−	-	no data	
RL9	−/−	-	no effect	
RL10	−/−	-	no effect	RL11
RL11	cyRL11/rh05	IgG Fc-binding glycoprotein	no effect	RL11
RL12	−/−	Putative membrane glycoprotein	no effect	RL11
RL13	−/−	Putative membrane glycoprotein	no effect	
RL14	−/−	-	no data	
UL1	−/−	-	no data	RL11
UL2	−/−	-	modest effect	RL11
UL3	−/−	-	no effect	
UL4	−/−	-	no effect	RL11
UL5	−/−	-	no effect	RL11
UL8	−/−	-	no effect	RL11
UL10	−/−	Temperance factor in retinal tissue^b^	no effect	RL11
UL12	−/−	-	modest effect	
UL15	−/−	-	no effect	
UL16	−/−	Membrane glycoportein involved in inhibiting Natural Killer cell cytotoxicity	no effect	
UL17	-/rh35	7-transmembrane glycoprotein	no effect	
UL18	−/−	MHCI homologue putative membrane protein	no effect	UL18
UL22	−/−	-	no data	
UL39	−/−	-	no effect	
UL40	-/rh67	Membrane glycoptrotein	no data	
UL58	−/−	-	no data	
UL59	−/−	-	no effect	
UL60	−/−	-	required for replication	
UL61	−/−	-	no data	
UL62	−/−	-	no effect	
UL63	−/−	-	no data	
UL64	−/−	-	no effect	
UL65	−/−	-	modest effect	
UL66	−/−	-	no data	
UL67	−/−	-	no effect	
UL68	−/−	-	no data	
UL80.5	-/rh109.1	Capsid Scaffold Protein	no data	Core
UL81	−/−	-	no data	
UL101	−/−	-	required for replication	
UL106	−/−	-	no data	
UL107	−/−	-	no data	
UL108	−/−	-	no data	
UL109	−/−	-	modest effect	
UL110	−/−	-	no effect	
UL118	-/rh151	-	no effect	
UL124	-/rh156.2	Membrane glycoprotein latent protein	no data	
UL125	−/−	-	variable critical effect	
UL127	−/−	-	no data	
UL129	−/−	-	no effect	
UL143	−/−	Inhibits Natural Killer cell cytotoxicity	modest effect	
UL142	−/−	Putative membrane protein	no data	
UL139	−/−	Putative membrane glycoprotein	no data	
UL138	−/−	Putative membrane protein	no data	
UL137	−/−	-	no data	
UL136	−/−	Putative membrane protein	no data	
UL135	−/−	Putative secreted protein	no data	
UL134	−/−	-	no data	
UL133	−/−	Putative membrane protein	no data	
UL148A	−/−	Putative membrane protein	no data	
UL148B	−/−	Putative membrane protein	no data	
UL148C	−/−	Putative membrane protein	no data	
UL148D	−/−	Putative membrane protein	no data	
UL149	−/−		no data	
UL150	−/−	Putative Secreted Protein	no data	
IRS1	−/−	Immediate early membrane protein & transcriptional activator/blocks protein kinase R mediated repression of translation	no data	
US4	−/−	-	no effect	US22
US5	-/rh183	-	no data	
US6	-/rh185	Putative membrane glycoprotein/Inhibits TAP mediated ER peptide transport	no data	
US7	−/−	Membrane glycoprotein	no effect	US6
US8	/-rh187	MHCI binding Membrane glycoprotein	no effect	US6
US9	−/−	Membrane glycoprotein involved in cell to cell spread	no effect	US6
US10	−/−	membrane glycoprotein/Delays trafficking of MHCI	no effect	US6
US15	−/−	Putative multiple transmembrane protein	no effect	US6
US16	−/−	Temperance factor and Putative multiple transmembrane protein	no effect	US12
US25	−/−	-	no effect	US12
US27	−/−	Virion Envelope Glycoprotein/Potentiates CXCR4 receptor^c^	no effect	
US33	−/−	-	no effect	GPCR
US34	−/−	Putative secreted protein	no effect	
US34A	−/−	Putative membrane protein	no effect	

Footnotes:

^a^Function annotated based on studies of HCMV [52] unless otherwise indicated

^b^Based on study of gene mutation in HCMV Towne [51]

^c^Based on study of US27 in HCMV AD169 [53]

### Anomalous gene trees reveal patterns of gene family evolution

Analysis of individual genes reveals several interesting features of these viral genomes. The Bayesian phylogeny for the important viral surface glycoprotein B gene (*UL55*), for example, indicates an atypically high level of divergence among these strains as compared to the whole genomes (Fig. 2b). This is of interest since it is a target of immune responses. In the analysis of the *UL55* gene, CyCMV Mauritius and CyCMV Ottawa do not cluster together, and CyCMV Mauritius instead is inferred to be more closely related to the RhCMVs than to CyCMV Ottawa. CyCMV Mauritius *UL55* is diverged from CyCMV Ottawa by 0.408 sps but diverged from RhCMV 68–1 and RhCMV 180.92 by only 0.175 and 0.188 sps respectively. Figure 2c shows a phylogenic estimate for the *US11* gene, which encodes an MHC down regulatory protein essential for superinfection [29]. Included in the analysis are multiple gene homologs of US11 generated by gene duplication in various CMVs. Inferred evolutionary relationships within US11 paralogs again indicate a closer evolutionary relationship between CyCMV Mauritius and the RhCMVs than to CyCMV Ottawa, although with weak statistical support (87.7 % posterior probability). Interestingly, rh188 is not present in RhCMV 180.92.

Figure 2d shows results of a phylogenetic analysis of the multiple copies of *US28*, a macaque CMV capsid protein that induces COX-2 in target cells upon entry. CyCMV Mauritius genes were named following the synteny-based system established for CyCMV Ottawa [18]. The five copies of CyCMV Ottawa, CyCMV Mauritius and RhCMV 180.92, as well as the four copies and single pseudogene of RhCMV 68–1, cluster first by synteny and then by species. This likely indicates that divergence of all five copies occurred before speciation and that loss of one copy in RhCMV 68–1 occurred after speciation. Unlike the other *US28* genes, however, phylogenetic sequence analysis of *US28e* (*rhUS28*) in RhCMV180.92, rh220 in RhCMV 68–1, *CyUS28e* (in both CyCMVs) suggests that CyCMV Mauritius and CyCMV Ottawa are each more closely related to RhCMV *US28e* than to CyCMV *US28e*. CyCMV Mauritius is diverged from CyCMV Ottawa by 0.175 sps and diverged from RhCMV 180.92 and RhCMV 68–1 by 0.031 sps and 0.031 sps, respectively. CyCMV Ottawa is diverged from RhCMV 180.92 and RhCMV 68–1 by 0.168 sps and 0.169 sps, respectively.

**Fig. 6:**
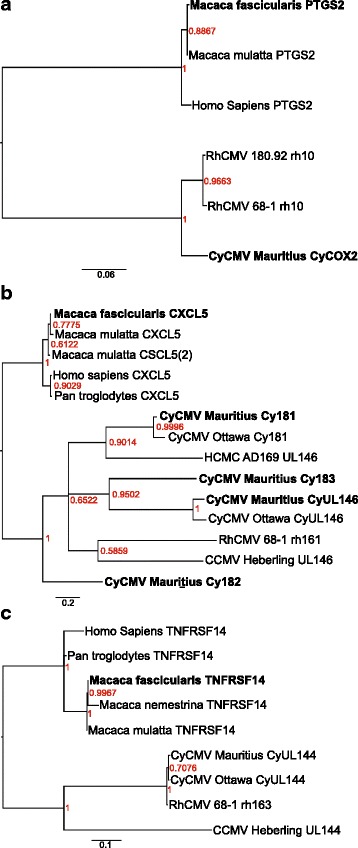
Phylogenetic tree comparison of CMV genes with mammalian homologues. Trees were prepared comparing CyCMV Mauritius, CyCMV Ottawa, RhCMV 68–1, RhCMV 180.92, CCMV Heberling and HCMV AD169 with mammalian homologue genes using the relevant *Homo sapiens* homologue as the outgroup. Trees were generated by Mr. Bayes following MAFFT alignment using a model of evolution selected by JModel test comparing **a**) CyCOX-2*/rh10* putative protein product to its mammalian homologue Prostaglandin-endoperoxide synthase 2 (*PTGS2*); **b**) *UL146* and *UL146*-like CMV genes with mammalian homologue CXC chemokine ligand 5 (*CXCL5*); **c**) *UL144* with mammalian homologue tumor necrosis factor receptor superfamily 14 (*TNFRSF14*). Numbers at loci indicate posterior probability with color scaled according to probability; genetic distances measured in substitutions per site (sps) are given by scale below

Phylogenetic relationships were further estimated for several CMV genes known to have homology to mammalian host genes (Fig. 6). Strain specific absence of several human and rhesus homologues supports independent evolution of each strain. This is evidenced, for example, by the absence of the *COX-2* gene in CyCMV Ottawa. Similarly, a COX-2 homologue (*rh10*, *CyCOX2*, this study), which promotes the formation of arachidonic acid in infected cells [30], is present in CyCMV Mauritius, RhCMV 180.92 and RhCMV 68–1, but absent in CyCMV Ottawa [30]. Viral COX-2 (*rh10*) is necessary for the infection of endothelial cells [30] and it has been shown that viral COX-2, but not cellular COX-2, protein is expressed when RhCMV 68–1 infects cells [30]. Since CMV has been implicated in vascular inflammation [31–33], the study of *rh10*, which confers cellular tropism and its selection in macaques, is of particular interest. *US28* is a viral capsid protein capable of inducing host COX-2 expression in target cells [34]. CyCMV Mauritius, CyCMV Ottawa and RhCMV 180.92 have five copies each while RhCMV 68–1 has only four copies of *US28* genes and a *US28* pseudogene. The pseudogene in RhCMV 68–1 shows that it, similar to other macaque CMVs, had five copies of *US28*, and that the fifth copy was likely pseudogenized after speciation. The number of copies of *US28* is variable across mammalian CMVs. Baboon CMV, similar to macaque CMV, has four distinct copies, HCMV has a single copy and both RCMV and MCMV lack *US28* homologues [35, 36]. These multiple copies are more divergent from each other within a genome than they are from corresponding homologues between macaque species (Fig. 2d) and can be assumed to have emerged prior to the speciation of these macaques.

COX-2 homologues are absent from CyCMV Ottawa and HCMV but present in both RhCMV 68–1 and RhCMV 180.92 [18, 19, 30, 34] Interestingly, however, the CyCMV Mauritius genome has three ORFs with homology to COX-2: *CyCOX-2A*, *CyCOX-2B*, and *CyCOX-2C*. It is perhaps not coincidental that RhCMV 68–1, which has a COX-2 homologue the least diverged from mammalian COX-2, is also the strain with fewest functioning *US28* genes. Meanwhile, a high degree of divergence between COX-2 homologues in the three strains that retain it suggests a more recent integration or only a low fitness advantage. The absence of the COX-2 homologue in CyCMV Ottawa and the fact that RhCMV 68–1 and CyCMV Mauritius are genetically less distant than RhCMV 68–1 and CyCMV Ottawa may again suggest a faster rate of divergence of CyCMV Ottawa. Comparison of COX-2 homologues (vCOX-2) showed a clustering of CMV homologues that are substantially diverged from their macaque homologs, prostaglandin endoperoxide synthase 2 (*PTGS2*) in *Macaca fascicularis* and *Macaca mulatta* (Fig. 6a). There is no HCMV equivalent of *PTGS2*. It can be inferred that the *COX-2* gene was copied from a mammalian host once to an ancestral CMV prior to CyCMV and RhCMV speciation. A mammalian origin of the vCOX-2 genes is further evidenced by the presence of introns in these genes. Perhaps the unique MHC haplotypes of Mauritian macaques favor the retention of an immune-modulatory gene, such as COX-2 homologue in CyCMV Mauritius.

Similarly, mammalian CXC chemokine ligand 5 (*CXCL5*) genes substantially diverged from CMV encoded *UL146*-like genes (Fig. 6b). Interestingly, viral chemokine-encoding *UL146*-like genes such as CCMV *UL146* clustered with RhCMV rh161, whereas HCMV *UL146* clustered with CyCMV *Cy181* with weak support. Two interesting observations were noted. First, both CCMV *UL146* and HCMV *UL146* are more diverged from mammalian *CXCL5* genes (closest is 2.075 sps and 2.069 sps respectively) than they are from multiple macaque CMV genes (closest are 1.299 sps and 1.870 sps respectively). Second, the CyCMV Mauritius gene Cy182 is less diverged from all the mammalian *CXCL5* genes than it is from any CMV genes (the closest mammalian gene is *Macaca mulatta CXCL5* at 1.234 sps, and the closest macaque gene is CyCMV Mauritius Cy181 1.448 sps). This probably reflects variation in the rate of evolution among these genes and among CMV strains, with the CyCMV Mauritius being slower than the others (Fig. 6b). The clustering of CCMV and HCMV with other CMVs may indicate the acquisition of a viral *CXCL5* homologue in a common ancestral CMV strain followed by gene duplication and mutation to create the UL146-like genes. This phylogeny supports at least two independent gene duplication events before diversification of Old World Primates. Possibly after this, other gene duplication events generated additional gene copies in macaques. Alternatively, more than two gene duplication events occurred in the ancestor of Old World primates followed by more extensive gene loss in apes than macaques. It is interesting that no *UL146*-like genes were identified in RhCMV180.92. Figure 6c shows a well supported close relationship between CMV UL144 genes of CyCMV Mauritius, CyCMV Ottawa, RhCMV 68–1 and CCMV Heberling to the exclusion of the homologous tumor necrosis factor receptor superfamily 14 (TNFRS14) genes in their macaque and human hosts, which is again consistent with a single copying event in the ancestor of these CMV strains.

### CMVs and their hosts share similar evolutionary patterns

**Fig. 7:**
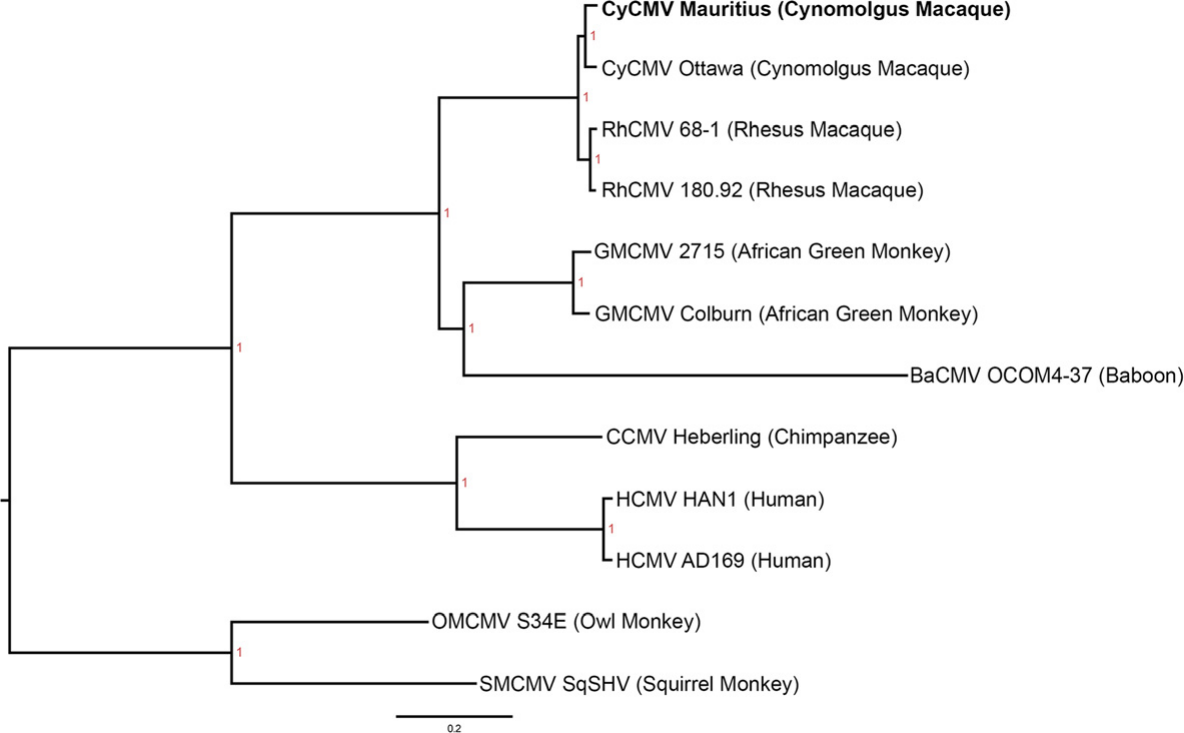
Bayesian phylogenetic tree of selected CMV strains. Generalized time reversible (GTR) tree with proportion invariant sites and gamma distribution of multiple sequenced primate CMV strains. Whole genome sequences were aligned using MAFFT with scoring matrix of 200PAM/k = 2 a gap open penalty of 1.53 and offset value of 0.123. New world primates, Owl Monkey (OMCMV) S34E and Squirrel Monkey (SMCMV) SqSHV were used as the out-group to root the tree. RhCMV 68–1, RhCMV 180.92, CyCMV Ottawa, CyCMV Mauritius, Chimpanzee CMV (CCMV) Heberling, Human CMV (HCMV) AD169, Human CMV (HCMV) HAN1, African Green Monkey CMV (GMCMV) Colburn and 2715, and Baboon CMV (BaCMV) OCOM4-37 were compared. Numbers at loci indicate posterior probability with color scaled according to probability; genetic distances measured in substitutions per site (sps) are given by scale below

In order to test for co-evolution between host species and the CMVs that they harbor over a broader phylogeny, we performed a phylogenetic analysis of 12 available primate CMV viral genomes (Fig. 7). The resulting phylogenetic tree of these CMVs was then compared to a previously estimated phylogenetic tree of mammalian genomes [37]. Phylogenetic relationships among multiple primate CMVs exhibit an identical pattern of CMV and host diversification (Fig. 7); [37]. There is evidence of attenuation in some sequenced CMVs caused by growth of the isolated strains in tissue culture prior to sequencing. This can result in the mutation or loss of viral genes necessary for entry or growth in certain cell types, as is the case with the deletion of the *UL128*-*UL130* region in RhCMV 68–1 [38], and other laboratory adapted CMV strains [39, 40]. Despite this, there is no indication of cross species contact or attenuation of some strains during CMV diversification prior to laboratory isolation. DNA polymerase sequence comparisons have suggested a speciation for CyCMV Ottawa and RhCMV 68–1 of around 0.5 mya [7]. This places speciation and divergence from a common CMV strain at ~1.2 mya, in line with Y-DNA segregation and the suspected end to macaque migration between islands of the Sunda shelf [41, 42].

It is possible for multiple CMV strains to co-exist within a single macaque, or within a single macaque population, because CMV can superinfect seropositive individuals [43]. However, co-infection of a host may only be possible for closely related CMV strains – cynomolgus macaques, for example, are not susceptible to infection or co-infection by a RhCMV [17]. The correspondence between viral and host evolutionary relationships suggests further study of CMV would serve as a molecular tool to understand primate evolution.

## Conclusions

In this study, we generated a novel genome sequence from a CMV strain isolated from a cynomolgus macaque from Mauritius. When analyzed with other CMV genomes from macaques and from other host species, inferred phylogenetic relationships among the viruses generally matched those among their hosts. Comparisons of this new genome with other macaque CMVs identify several functional categories of genes with atypically high levels of divergence, variation in gene content, and several genes with inferred phylogenetic relationships that differed from the genome-wide estimate. These results have implications for use of CMV as a vaccine vector and molecular tool, in CMV pathogenesis studies, as well as providing a tool to assist in tracing both viral and macaque migration and distribution.

## Methods

### Isolating virus

Ethics approval was obtained for all animal procedures through the Health Canada National Nonhuman Primate Animal Care Committee. Urine samples were collected from a 15 year-old cynomolgus macaque imported from Mauritius using bladder catheterization. After filtration through a 0.45 μm filter, the urine samples were centrifuged at 900 x *g* for 30 mins at 4 °C and supernatants were collected and mixed 1:1 with 2X MEM (supplemented with 2X antibiotic-antimycotic and 20 mg/ml gentamycin; Invitrogen). The cell pellets were resuspended with 1 ml of Dulbecco’s Modified Eagle’s Medium (DMEM; Sigma) supplemented with 1X antibiotic-antimycotic (Gibco), and 10 mg/ml gentamycin (Invitrogen). Urine supernatants were ultracentrifuged at 20 000 x g for 30 mins at 4 °C and the pellets resuspended in 500 μl of DMEM (supplemented with 1X antibiotic-antimycotic and 10 mg/ml gentamycin).

CyCMV Mauritius virus was grown on human fetal lung fibroblast (MRC-5) cells [44] from the American Type Culture Collection (ATCC) as described previously [18]. MRC-5 cells were seeded 1:2 in a 12-well tissue culture plate and grown at 37 °C in a 5 % CO_2_ incubator for 2 days (until the cell confluence reached near 100 %) prior to inoculation. Resuspended viral pellets were each plated in triplicate in wells of MRC-5 cells, spinoculated at 2000 x *g* for 30 mins at 4 °C, and incubated for 2–3 h at 37 °C in a 5 % CO_2_ incubator. The inoculum was aspirated and replaced with 2 ml of DMEM [supplemented with 10 % FBS (Wisent Bioproducts), 100 U/ml penicillin, 100 μg/ml streptomycin (Sigma)] and amphotericin B. The medium was changed the following day and every week thereafter. Cultures were monitored daily for CMV cytopathic effect (CPE). Cells showed 100 % CPE after approximately 1 month. The virus was further propagated in MRC-5 cells at a 1:2 split ratio each time CPE reached 90–100 % to achieve higher viral titers.

### DNA extraction

Viral DNA was isolated from cell free virus following centrifugation, digestion with pronase, and phenol-chloroform extraction. Fifteen 75-cm^2^ tissue culture flasks containing CyCMV Mauritius-infected MRC-5 cells were used for viral DNA isolation using a Hirt extraction protocol as described previously [18]. To confirm the purity of the viral DNA isolation, 1 μg of isolated extracellular viral DNA was digested with 20 U of *BamH*I or *Hind*III restriction enzymes (New England Biolabs) and fractionated by gel electrophoresis on a 0.8 % agarose gel.

### Next Generation Sequencing (NGS)

A paired-end library of CyCMV Mauritius DNA was prepared with 500 bp insert size and high-throughput Illumina Genome Analyzer II paired end sequencing with 72 bp read length was performed as described previously [18]. The complete genome was sequenced at the Centre of Applied Genomics, Toronto, Canada.

### Genome assembly

*De novo* assembly of the CyCMV Mauritius genome was achieved from 2 268 822 reads on the Illumina Genome Analyzer II platform. Paired ends were filtered to match the barcode (1 134 411 paired reads) and assembled using Velvet (version 0.7.55) [45]. Results were best obtained using a kmer length of 39, an insert length of 500, and an expected coverage of 280X to generate multiple partial contigs. Three seed sequences, A: 5’-AAACCAGCGCCGTTGTTTTCCGTTCTACGTTCGGG-3’, B: 5’-ACTATCGAGGACAACGATGTTTTTTCCAACATAAA-3’ and C: 5’-TATCGGTATCTATTCCAAGCAGACCAAGTACGATT-3’ were used to create contigs.

### Gap closing

Gap closing in the *de novo* genome build was accomplished using PCR. Primers were designed from the sequenced contigs, and amplified using purified viral DNA. The resulting product sizes were used to confirm the *de novo* DNA assembly.

### Error correction

Regions of low resolution or ambiguity were confirmed using Sanger sequencing. First, a 3 555 bp region (3 567–7 122 bp) was confirmed using the primers 5’-TCGGCAAAGTCAGGAGCGGC-3’ and 5’-TGCACAATTTGCGATGCCTATCGTT-3’. Next, Sanger sequencing to amplify the 1 207 bp region spanning the origin of lytic replication (OriLyt) [82 561 bp–83 768 bp] was attempted using the primers 5’-TGGCGATCTGAAACCACACCCC-3’ and 5’-CGCCCAAGAGAGAGCGCACC-3’ but proved challenging due to the presence of inverted and repeated sequence motifs [46] as our previous experience has shown [18]. Amplification of a 1 688 bp segment spanning 154 169 bp -155 856 bp using multiple primers also proved unsuccessful. However, a 560 bp region spanning 173 964 bp - 174 520 bp was amplified using the primers 5’-ACTTCGCTTCTGTTCTAGCGTTTAGG-3’ and 5’-CCGCTGTGGCTTGCTGGCTC-3’ and successfully confirmed by sequencing. The CyCMV Mauritius sequence was finally confirmed for errors by aligning with that of RhCMV 68–1 (accession: AY186194), RhCMV 180.92 (accession: DQ120516) and CyCMV Ottawa (accession: JN227533).

### Open Reading Frame (ORF) assignment

1408 putative ORFs, greater than 30 amino acids in length, and not contained within and in frame of another identified potential ORFs were identified *in silico* using Geneious Pro 6.1.4 (Biomatters Ltd., Auckland, New Zealand). BLAST-P (NCBI) with a BLOSUM62 matrix, gap opening cost of 11 and gap extension cost of one was used to screen the ORFs. Homologous protein lists were generated using a maximum E value of 10^−1^. ORFs with irrelevant or no homology were not included.

### Nomenclature

Putative genes were named with respect to homologues, as determined above, when applicable. Homologues of human genes are given the prefix ‘Cy’ followed by the names, terminal region long (TRL), terminal region short (TRS), unique long (UL), or unique short (US) and a number previously attributed to the homologue. Homologues of old world monkey CMV genes were given the prefix ‘Cy’ followed by ‘O’ and a gene number attributed to the homologue. Some of these were given alternative names, indicated in brackets, due to naming of CyCMV Ottawa homologues prior to this nomenclature [18]. Putative genes with previously named CyCMV Ottawa homologues were given the prefix ‘Cy’ followed by the previously assigned number. Putative genes lacking a CMV homologue were given the prefix ‘Cy’ followed by a unique number not used in naming within CyCMV Ottawa and corresponding to gene order within the genome. Capital letter suffixes (for example, *CyUL48***A**) indicate unique ORFs with a shared gene number as previously established [18]. Lower case single letter suffixes (for example CyUS28**b**) indicate closely related or repeated genes that share homologous partners. Putative genes were assigned to one of 18 families, or designated non-core (without family), based on previously designated family of gene homologues.

### Bit-score plots

Bit-scores were calculated using Geneious Pro 6.1.4 (Biomatters Ltd., Auckland, New Zealand) to compare individual CyCMV Mauritius or RhCMV 180.92 ORFs to homologous ORFs of CyCMV Ottawa (accession: JN227533), RhCMV 180.92 (accession: DQ120516), and RhCMV 68–1 (accession: AY186194) found using BLAST-P (NCBI) using a BLOSUM62 matrix, gap opening cost of 11, gap extension cost of one and a maximum E value of 10^−1^. Calculated scores for individual ORF were plotted as seen previously [18] as a scatterplot comparing the bit-score for CyCMV Mauritius-CyCMV Ottawa, CyCMV Mauritius -RhCMV 180.92 and CyCMV Mauritius -RhCMV 68–1 to each other.

### Gross genome comparison

CyCMV Mauritius has been deposited in the GenBank database (accession: KP796148). Other Genomes and annotations were obtained from GenBank using available strains as follows: CyCMV Ottawa (accession: JN227533), RhCMV 180.92 (accession: DQ120516), and RhCMV 68–1 (accession: AY186194). Comparisons were also made with human CMV strains [HCMV Towne (accession: AY315197), HCMV AD169 (accession: X17403) and HCMV HAN1 (accession: JX512199)], chimpanzee CMV Heberling (accession: AF480884), Baboon cytomegalovirus OCOM4-37 (accession: AC090446), Cercopithecine herpesvirus 5 (GMCMV) Colburn (accession: FJ483969), Cercopithecine herpesvirus 5 (GMCMV) strain 2715 (accession: FJ483968), Aotine herpesvirus 1 strain S34E (accession: FJ483970), and Saimiriine herpesvirus 3 SqSHV (accession: FJ483967). Genome identity between CMV strains was determined using global alignment with free end gaps set at a cost matrix of 65 % similarity (5.0/-4.0), a gap open penalty of 12 and a gap extension penalty of three with automatic sequence direction determination on Geneious 6.1.4 (Biomatters Ltd., Auckland, New Zealand). Whole genome alignments were generated using progressive MAUVE multiple genome alignment algorithm [21] available in Geneious 6.1.4 (Biomatters Ltd., Auckland, New Zealand).

### Phylogenetic analysis

Phylogenetic analysis was carried out with Geneious Pro 6.1.4 (Biomatters Ltd., Auckland, New Zealand). Alignments were created using MAFFT version 7.017 [47] with a scoring matrix of 200PAM/k = 2 a Gap open penalty of 1.53 and offset value of 0.123. An appropriate phylogenetic model was selected for each tree using JModel test 2.1.7 [48]. Most trees were generated with the MrBayes plugin for Geneious Pro 6.1.4 [49] after 1 100 000 iterations and a burn-in of 110 000. The individual ORFs or whole genome sequences of CyCMV Mauritius, CyCMV Ottawa (accession: JN227533), RhCMV 68–1 (accession: AY186194), and RhCMV 180.92 (accession: DQ120516), were compared with the outgroup HCMV AD169 (accession: FJ527563) (Fig. 2), Homo Sapiens PTGS2 (accession: BAA05698) (Fig. 6a), Homo Sapiens CXCL5 (accession: CR457428) (Fig. 6b), or Homo Sapiens TNFRSF14X1 (trimmed CDS from accession: XM_011542383) (Fig. 6c). The 12 CMV full genome tree (Fig. 7) was generated using MrBayes 3.2.4 run in MPI on the SciNet supercomputer at the University of Toronto [50] using a generalized time reversible (GTR) tree with proportion invariant sites and gamma distribution. Genomes utilized were selected from fully sequenced CMV genomes at the time of publication due to similar organization. CMVs included were free from large rearrangements or inverted regions with respect to CyCMV Mauritius as determined from alignments using progressive MAUVE multiple genome alignment algorithm [21] in Geneious 6.1.4 (Biomatters Ltd., Auckland, New Zealand). RCMV Maastricht (Rat) (accession: AF232689) was used as the out-group.

### Availability of data and materials

The genome sequence for CyCMV Mauritius has been deposited in the GenBank database (accession no. KP796148).

## Additional files

Additional file 1: Figure S1. Map of ORFs in CyCMV Mauritius genome. CyCMV Mauritius encodes 290 putative ORFs that are annotated by gene name and colour coded based on gene families. Of the CyCMV Mauritius ORFs, 268 (92 %) share homologues with CyCMV Ottawa, 239 share homologues with RhCMV 68–1 (82 %), and 158 (54 %) share homologs with HCMV strains. CyCMV Mauritius like RhCMV but unlike CyCMV Ottawa or HCMV contains ORFs with homology to COX-2. CyCMV ORFs with an HCMV homologue are annotated by “Cy” followed by the HCMV name. Arrowheads indicate the directions of the ORFs. Core genes are herpes virus core genes. (PDF 4584 kb) Additional file 2: Table S1. ORFs of CyCMV Mauritius. Footnotes: 1 Functions annotated based on studies of HCMV [52] unless otherwise indicated. 2 Nearest homologous HCMV gene based on Bit score using BLASTP search. Strain of HCMV gene indicated with footnote. 3 Based on function of RhCMV68-1 homologue [19]. 4 Based on function of CyCMV Ottawa homologue [18]. 5 Based on studies of HCMV Towne-BAC [54]. 6 Inferred through function of homologous proteins [52]. 8 HCMV strain AD169. 9 HCMV strain AF1. 10 HCMV strain ASM72. 11 HCMV strain C154. 12 HCMV strain C425, 13 HCMV strain CINCY + Towne. 14 HCMV strain Coz. 15 HCMV strain David. 16 HCMV strain FRCMV-14L. 17 HCMV strain GSV6. 18 HCMV strain GSV9. 19 HCMV strain HAN1. 20 HCMV strain HAN13. 21 HCMV strain HAN16. 22 HCMV strain HAN20. 23 HCMV strain HAN3. 24 HCMV strain HAN38. 25 HCMV strain HKS40. 26 HCMV strain I-10. 27 HCMV strain IS17. 28 HCMV strain JHC. 29 HCMV strain JP. 30 HCMV strain L2. 31 HCMV strain Merlin. 32 HCMV strain RK (Human Herpesvirus 7). 33 HCMV strain TB40/E. 34 HCMV strain Toledo. 35 HCMV strain Towne. 36 HCMV strain TR. 37 HCMV strain U11. 38 HCMV strain U8. 39 HCMV strain VR1814. 40 HCMV strain 35. 41 HCMV strain 66. 42 HCMV strain 452. 43 HCMV strain 553. 44 HCMV strain 3157. 45 HCMV strain 3301. 46 HCMV strain 5234. 47 HCMV strain 401058. 48 HCMV strain 26M. 49 HCMV strain 51C. (PDF 158 kb) Additional file 3: Figure S2. Break down of two-dimensional bit-score plots between CMV Mauritius-RhCMV 68.1 versus CyCMV Mauritius-RhCMV 180.92 by ORF function. Graphs represent comparisons of ORF homologue bit-scores across three strains simultaneously. ORFs are coloured according to known function. Individual ORF may have several functions and are present in multiple plots. Immune ORFs are involved in host immune regulation or evasion, entry ORFs are known cell entry factors, temperance ORFs are involved in temperance of viral growth, membrane ORFs are presented in cell membrane on infected cells or in virions, DNA/nuclear are known to interact with DNA or shuttle to the cell nucleus, capsid ORFs form the virus capsid, tegument proteins are found in the viral tegument, the function of ORFs with other known function vary, and the function is unknown at the time of writing for ORFs plotted as unknown. ORFs are annotated according to CyCMV Mauritius names except for ORFs of unknown function or membrane ORF where some ORF are left unlabelled. (PDF 49 kb) Additional file 4: Figure S3. Break down of two-dimensional bit-score plots between CyCMV Mauritius-CyCMV Ottawa versus RhCMV 68-1-RhCMV 180.92 by ORF function. Graphs represent comparisons of ORF homologue bit-scores across three strains simultaneously. ORFs are coloured according to known function. Individual ORF may have several functions and are present in multiple plots. Immune ORFs are involved in host immune regulation or evasion, entry ORFs are known cell entry factors, temperance ORFs are involved in temperance of viral growth, membrane ORFs are presented in cell membrane on infected cells or in virions, DNA/nuclear are known to interact with DNA or shuttle to the cell nucleus, capsid ORFs form the virus capsid, tegument proteins are found in the viral tegument, the function of ORFs with other known function vary, and the function is unknown at the time of writing for ORFs plotted as unknown. ORFs are annotated according to CyCMV Mauritius names except for ORFs of unknown function or membrane ORF where some ORF are left unlabelled. (PDF 52 kb) Additional file 5: Figure S4. Break down of two-dimensional bit-score plots between CyCMV Mauritius-CyCMV Ottawa versus CyCMV Mauritius-RhCMV 180.92 by ORF function. Graphs represent comparisons of ORF homologue bit-scores across three strains simultaneously. ORFs are coloured according to known function. Individual ORF may have several functions and are present in multiple plots. Immune ORFs are involved in host immune regulation or evasion, entry ORFs are known cell entry factors, temperance ORFs are involved in temperance of viral growth, membrane ORFs are presented in cell membrane on infected cells or in virions, DNA/nuclear are known to interact with DNA or shuttle to the cell nucleus, capsid ORFs form the virus capsid, tegument proteins are found in the viral tegument, the function of ORFs with other known function vary, and the function is unknown at the time of writing for ORFs plotted as unknown. ORFs are annotated according to CyCMV Mauritius names except for ORFs of unknown function or membrane ORF where some ORF are left unlabelled. (PDF 50 kb) Additional file 6: Figure S5. Break down of two-dimensional bit-score plots between CyCMV Mauritius-CyCMV Ottawa versus CyCMV Mauritius-RhCMV 68–1 by ORF function. Graphs represent comparisons of ORF homologue bit-scores across three strains simultaneously. ORFs are coloured according to known function. Individual ORF may have several functions and are present in multiple plots. Immune ORFs are involved in host immune regulation or evasion, entry ORFs are known cell entry factors, temperance ORFs are involved in temperance of viral growth, membrane ORFs are presented in cell membrane on infected cells or in virions, DNA/nuclear are known to interact with DNA or shuttle to the cell nucleus, capsid ORFs form the virus capsid, tegument proteins are found in the viral tegument, the function of ORFs with other known function vary, and the function is unknown at the time of writing for ORFs plotted as unknown. ORFs are annotated according to CyCMV Mauritius names except for ORFs of unknown function or membrane ORF where some ORF are left unlabelled. (PDF 50 kb) Additional file 7: Figure S6. Plot of bit-scores of ORFs across the CyCMV Mauritius genome. ORFs of CyCMV Mauritius are ordered as seen in CyCMV Mauritius genome. Bit-score is plotted for each ORF between CyCMV Mauritius and CyCMV Ottawa, RhCMV 68–1, and RhCMV 180.92. Absence of the indicated gene from one or more compared genomes results in a bit-score of zero. Values are indicated as the means ± standard deviation for each gene. (PDF 147 kb) Additional file 8: Figure S7. Break down of two-dimensional bit-score plots between CMV Mauritius-CyCMV Ottawa versus RhCMV 68-1-RhCMV 180.92 by ORF family. Graphs represent comparisons of ORF homologue bit-scores across three strains simultaneously. ORFs are coloured according to ORF family. CyCMV Mauritius names for ORF labels. (PDF 57 kb) Additional file 9: Figure S8. Break down of two-dimensional bit-score plots between CyCMV Mauritius-RhCMV 68–1 versus CyCMV Mauritius-RhCMV 180.92 by ORF family. Graphs represent comparisons of ORF homologue bit-scores across three strains simultaneously. ORFs are coloured according to ORF family. CyCMV Mauritius names for ORF labels. (PDF 56 kb) Additional file 10: Figure S9. Break down of two-dimensional bit-score plots between CMV Mauritius-CyCMV Ottawa versus CyCMV Mauritius-RhCMV 180.92 by ORF family. Graphs represent comparisons of ORF homologue bit-scores across three strains simultaneously. ORFs are coloured according to ORF family. CyCMV Mauritius names used for ORF labels. (PDF 57 kb) Additional file 11: Figure S10. Break down of two-dimensional bit-score plots between CMV Mauritius-RhCMV 68–1 versus CyCMV Mauritius-CyCMV Ottawa by ORF family. Graphs represent comparisons of ORF homologue bit-scores across three strains simultaneously. ORFs are coloured according to ORF family. CyCMV Mauritius names used for ORF labels. (PDF 57 kb)
