# Supplementary figures and images for: A novel strain of cynomolgus macaque cytomegalovirus: implications for host-virus co-evolution
Source: BMC Genomics. 2016 Apr 5;17:277. doi: 10.1186/s12864-016-2588-3 (PMC4820910; doi:10.1186/s12864-016-2588-3)

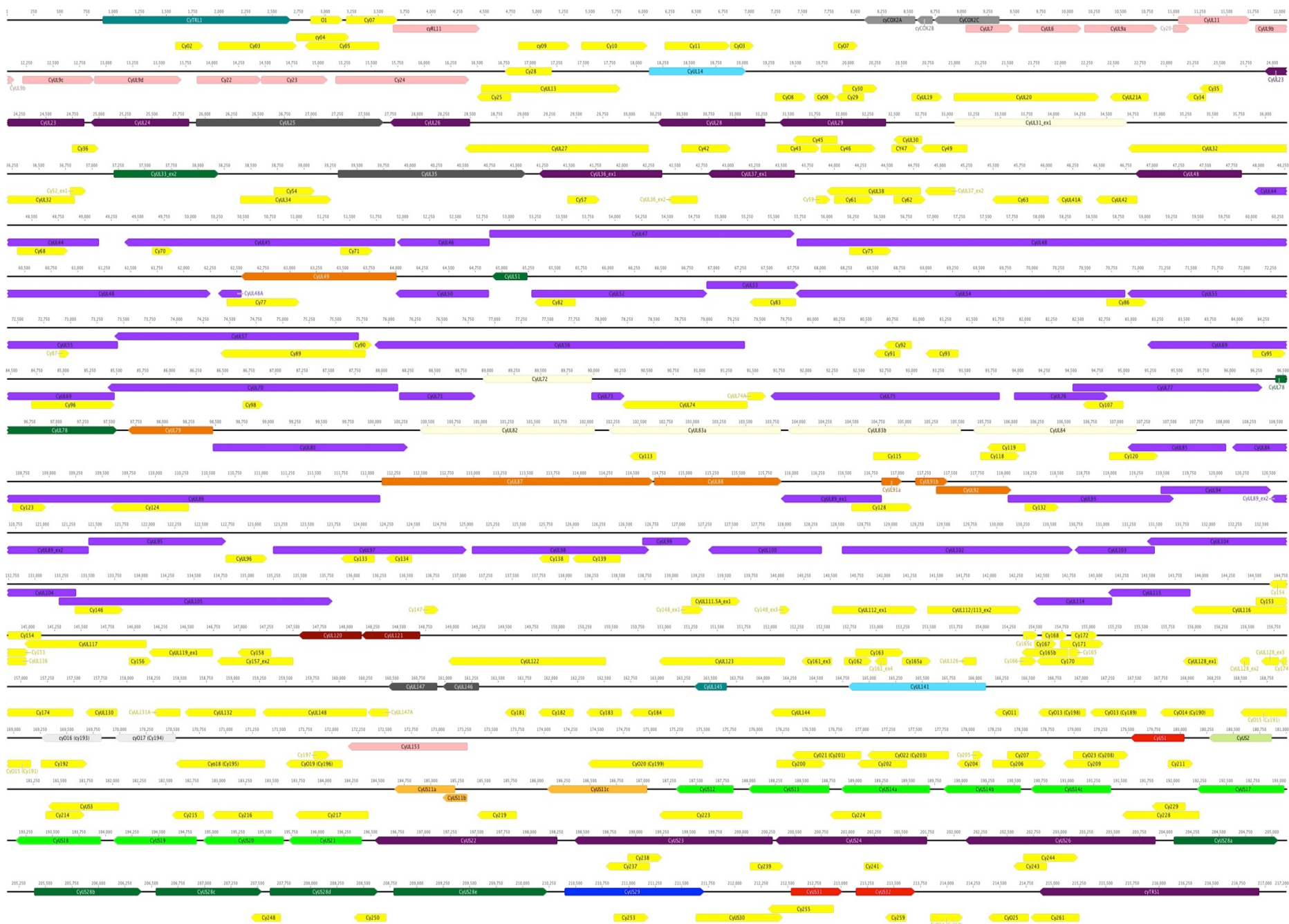

No family (170)    Core Gene (38)    CXCL (2)    dUTPase (6)    RL1 (2)    LL11 (13)    UL120 (2)    US1 (3)    US22 (13)    Cyclooxygenase-2 (3)    O16 (2)    US12 (10)    Betagama ORF (7)    GPCR (8)    UL14 (2)    US2 (1)    US25 (2)    US29 (1)    US6 (3)

Supplement: Additional file 1: Figure S1. — Map of ORFs in CyCMV Mauritius genome. CyCMV Mauritius encodes 290 putative ORFs that are annotated by gene name and colour coded based on gene families. Of the CyCMV Mauritius ORFs, 268 (92 %) share homologues with CyCMV Ottawa, 239 share homologues with RhCMV 68–1 (82 %), and 158 (54 %) share homologs with HCMV strains. CyCMV Mauritius like RhCMV but unlike CyCMV Ottawa or HCMV contains ORFs with homology to COX-2. CyCMV ORFs with an HCMV homologue are annotated by “Cy” followed by the HCMV name. Arrowheads indicate the directions of the ORFs. Core genes are herpes virus core genes. (PDF 4584 kb) [file 12864_2016_2588_MOESM1_ESM.pdf]

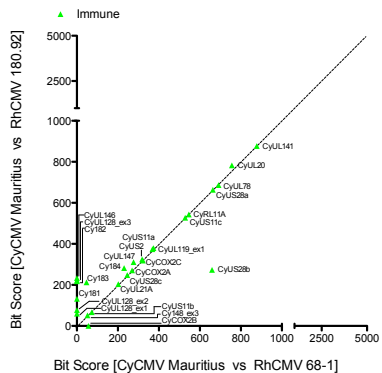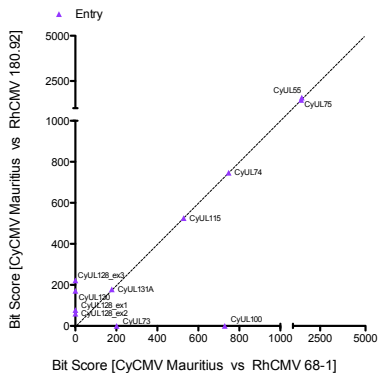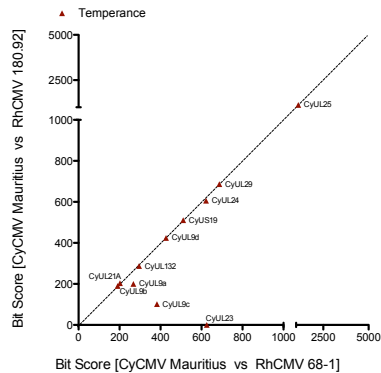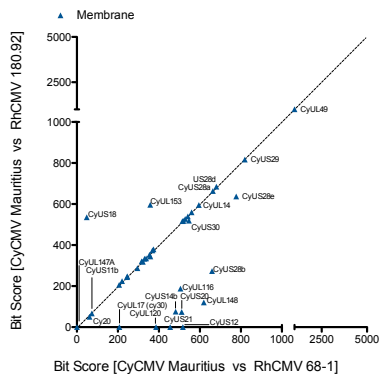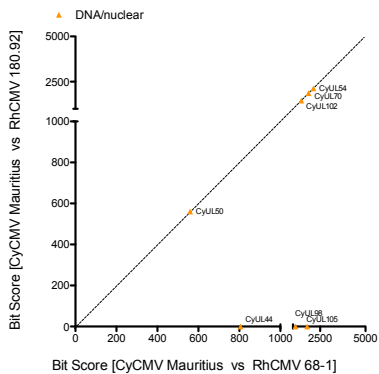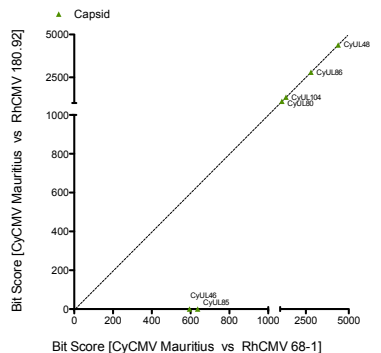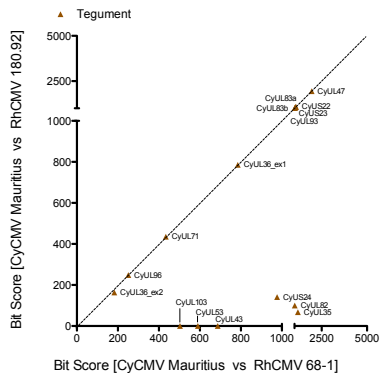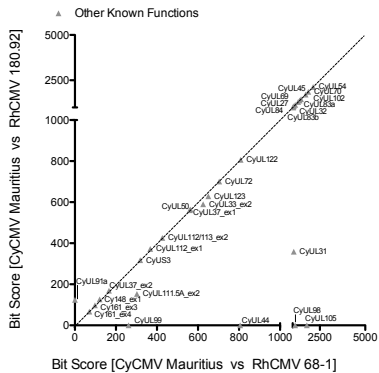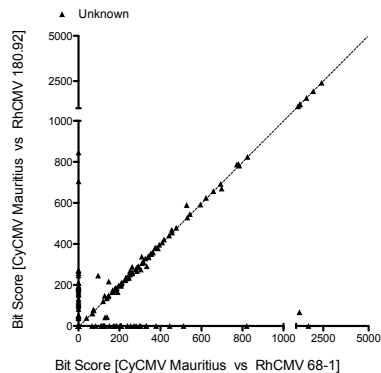

Supplement: Additional file 3: Figure S2. — Break down of two-dimensional bit-score plots between CMV Mauritius-RhCMV 68.1 versus CyCMV Mauritius-RhCMV 180.92 by ORF function. Graphs represent comparisons of ORF homologue bit-scores across three strains simultaneously. ORFs are coloured according to known function. Individual ORF may have several functions and are present in multiple plots. Immune ORFs are involved in host immune regulation or evasion, entry ORFs are known cell entry factors, temperance ORFs are involved in temperance of viral growth, membrane ORFs are presented in cell membrane on infected cells or in virions, DNA/nuclear are known to interact with DNA or shuttle to the cell nucleus, capsid ORFs form the virus capsid, tegument proteins are found in the viral tegument, the function of ORFs with other known function vary, and the function is unknown at the time of writing for ORFs plotted as unknown. ORFs are annotated according to CyCMV Mauritius names except for ORFs of unknown function or membrane ORF where some ORF are left unlabelled. (PDF 49 kb) [file 12864_2016_2588_MOESM3_ESM.pdf]

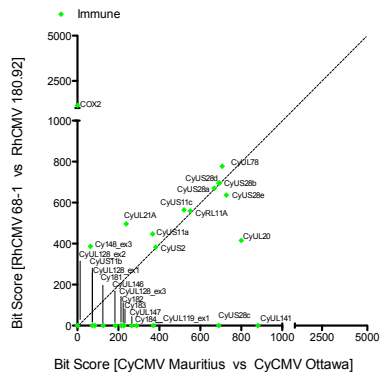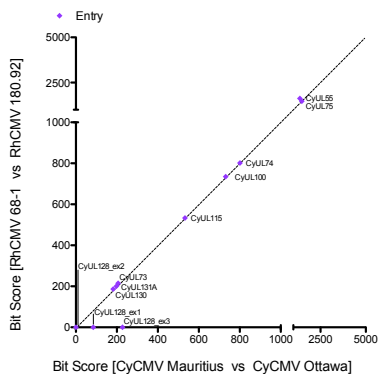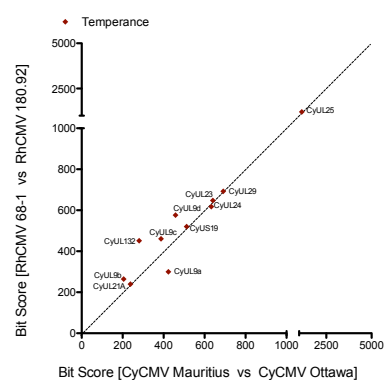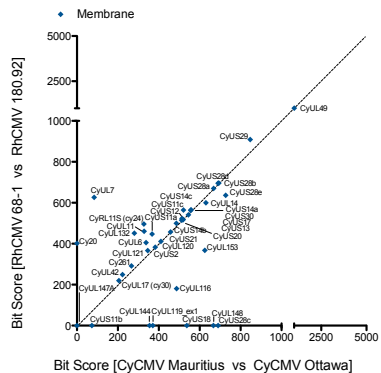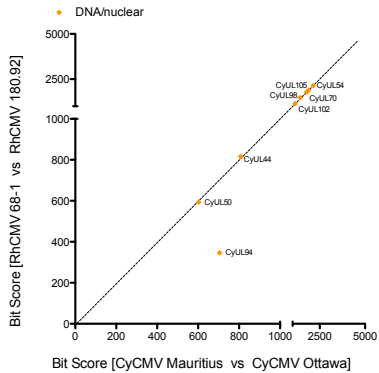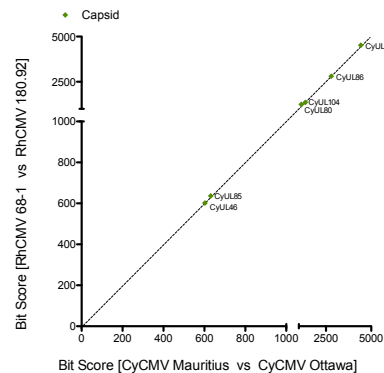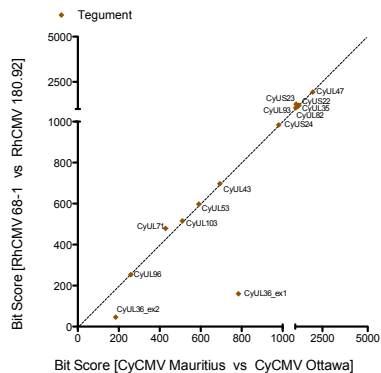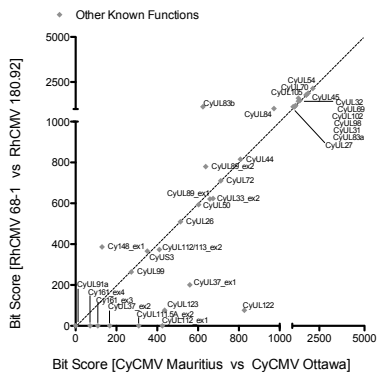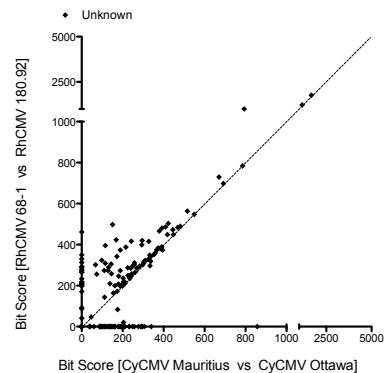

Supplement: Additional file 4: Figure S3. — Break down of two-dimensional bit-score plots between CyCMV Mauritius-CyCMV Ottawa versus RhCMV 68-1-RhCMV 180.92 by ORF function. Graphs represent comparisons of ORF homologue bit-scores across three strains simultaneously. ORFs are coloured according to known function. Individual ORF may have several functions and are present in multiple plots. Immune ORFs are involved in host immune regulation or evasion, entry ORFs are known cell entry factors, temperance ORFs are involved in temperance of viral growth, membrane ORFs are presented in cell membrane on infected cells or in virions, DNA/nuclear are known to interact with DNA or shuttle to the cell nucleus, capsid ORFs form the virus capsid, tegument proteins are found in the viral tegument, the function of ORFs with other known function vary, and the function is unknown at the time of writing for ORFs plotted as unknown. ORFs are annotated according to CyCMV Mauritius names except for ORFs of unknown function or membrane ORF where some ORF are left unlabelled. (PDF 52 kb) [file 12864_2016_2588_MOESM4_ESM.pdf]

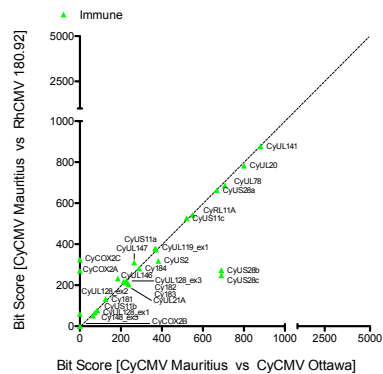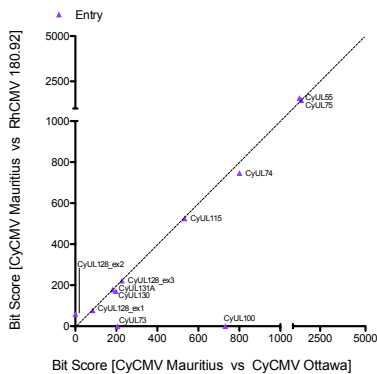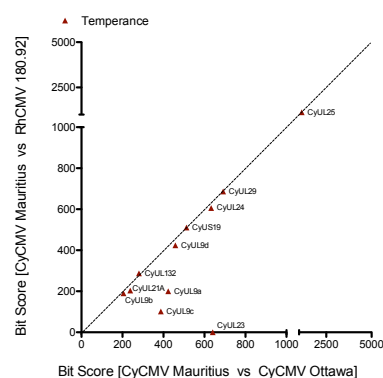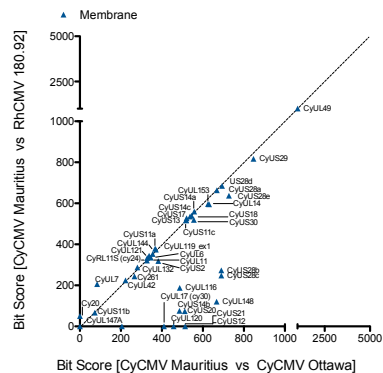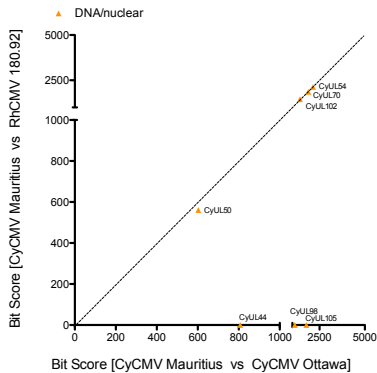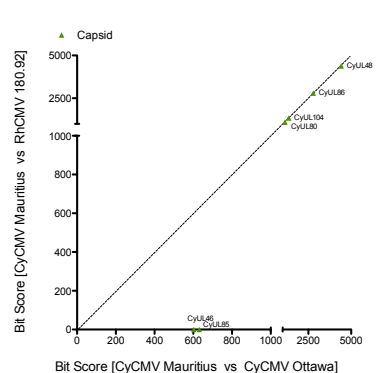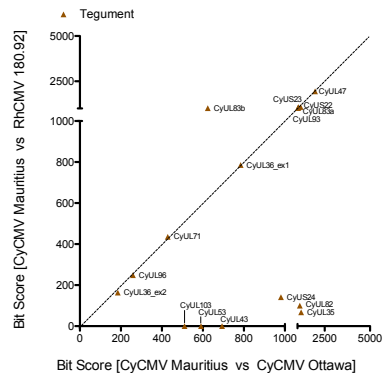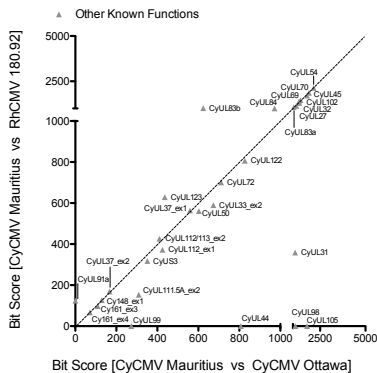

Supplement: Additional file 5: Figure S4. — Break down of two-dimensional bit-score plots between CyCMV Mauritius-CyCMV Ottawa versus CyCMV Mauritius-RhCMV 180.92 by ORF function. Graphs represent comparisons of ORF homologue bit-scores across three strains simultaneously. ORFs are coloured according to known function. Individual ORF may have several functions and are present in multiple plots. Immune ORFs are involved in host immune regulation or evasion, entry ORFs are known cell entry factors, temperance ORFs are involved in temperance of viral growth, membrane ORFs are presented in cell membrane on infected cells or in virions, DNA/nuclear are known to interact with DNA or shuttle to the cell nucleus, capsid ORFs form the virus capsid, tegument proteins are found in the viral tegument, the function of ORFs with other known function vary, and the function is unknown at the time of writing for ORFs plotted as unknown. ORFs are annotated according to CyCMV Mauritius names except for ORFs of unknown function or membrane ORF where some ORF are left unlabelled. (PDF 50 kb) [file 12864_2016_2588_MOESM5_ESM.pdf]

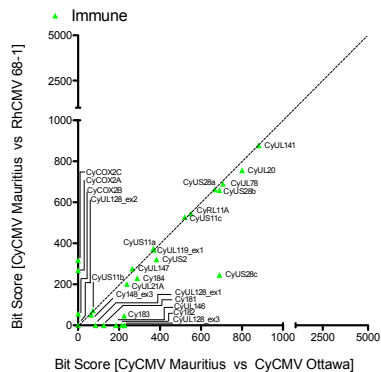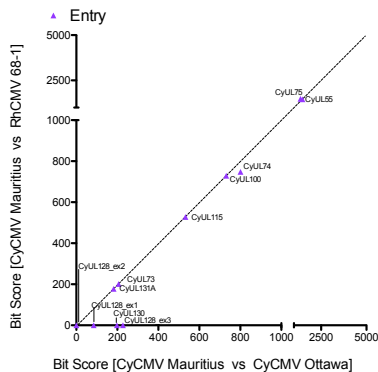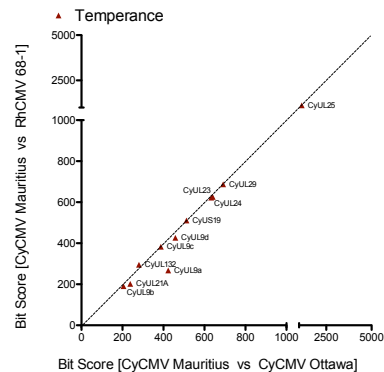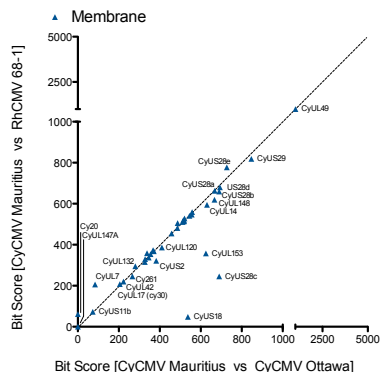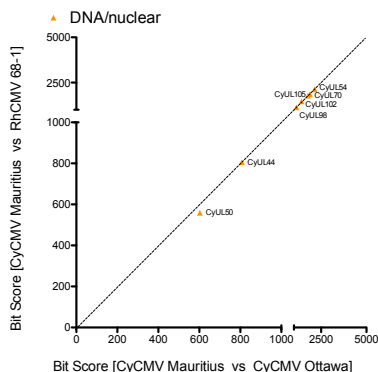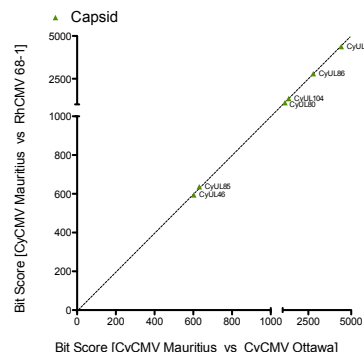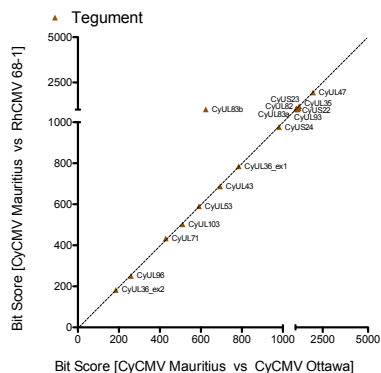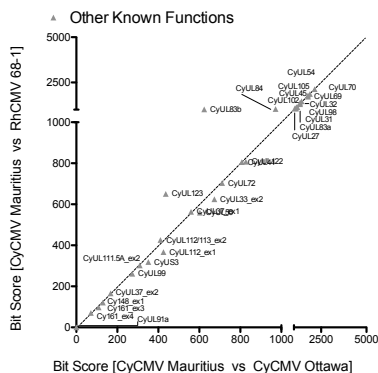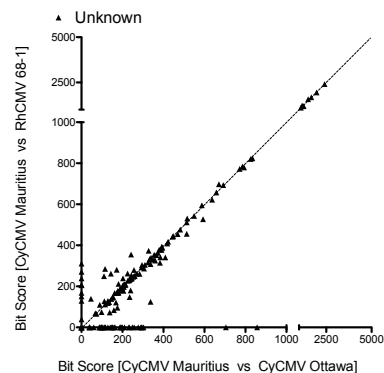

Supplement: Additional file 6: Figure S5. — Break down of two-dimensional bit-score plots between CyCMV Mauritius-CyCMV Ottawa versus CyCMV Mauritius-RhCMV 68–1 by ORF function. Graphs represent comparisons of ORF homologue bit-scores across three strains simultaneously. ORFs are coloured according to known function. Individual ORF may have several functions and are present in multiple plots. Immune ORFs are involved in host immune regulation or evasion, entry ORFs are known cell entry factors, temperance ORFs are involved in temperance of viral growth, membrane ORFs are presented in cell membrane on infected cells or in virions, DNA/nuclear are known to interact with DNA or shuttle to the cell nucleus, capsid ORFs form the virus capsid, tegument proteins are found in the viral tegument, the function of ORFs with other known function vary, and the function is unknown at the time of writing for ORFs plotted as unknown. ORFs are annotated according to CyCMV Mauritius names except for ORFs of unknown function or membrane ORF where some ORF are left unlabelled. (PDF 50 kb) [file 12864_2016_2588_MOESM6_ESM.pdf]

**a**

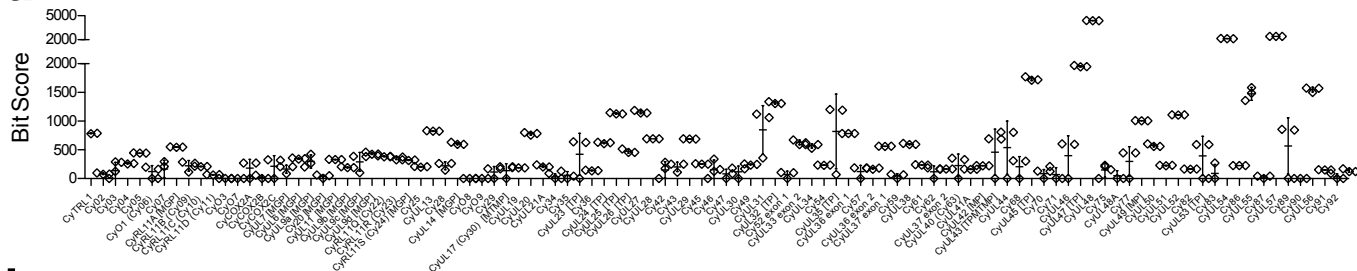

**b**

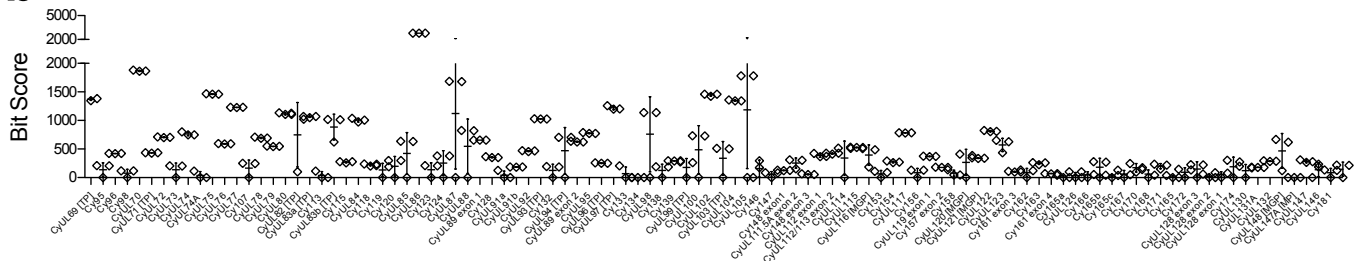

**C**

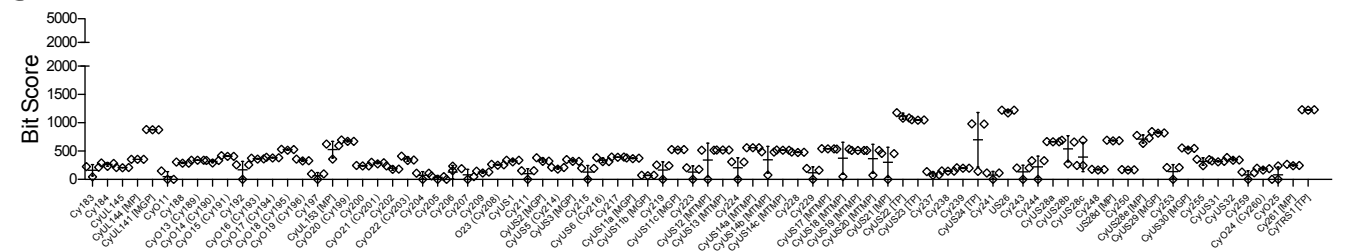

Supplement: Additional file 7: Figure S6. — Plot of bit-scores of ORFs across the CyCMV Mauritius genome. ORFs of CyCMV Mauritius are ordered as seen in CyCMV Mauritius genome. Bit-score is plotted for each ORF between CyCMV Mauritius and CyCMV Ottawa, RhCMV 68–1, and RhCMV 180.92. Absence of the indicated gene from one or more compared genomes results in a bit-score of zero. Values are indicated as the means ± standard deviation for each gene. (PDF 147 kb) [file 12864_2016_2588_MOESM7_ESM.pdf]

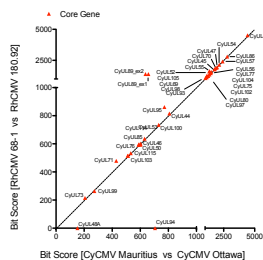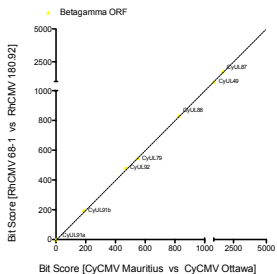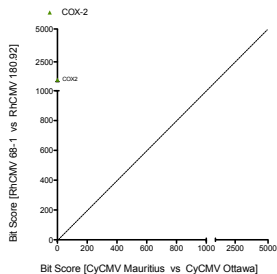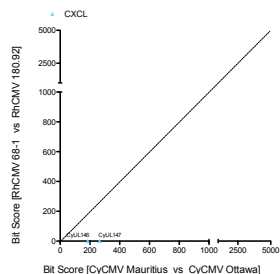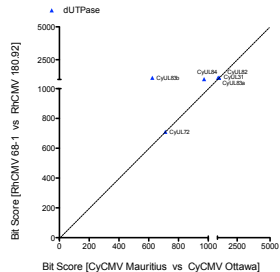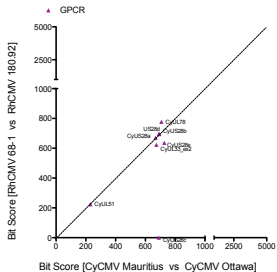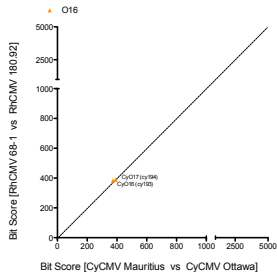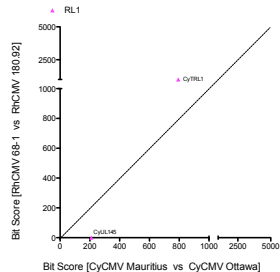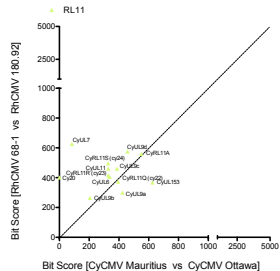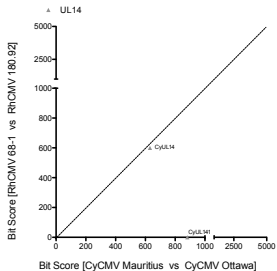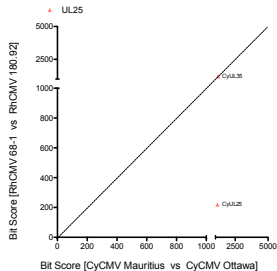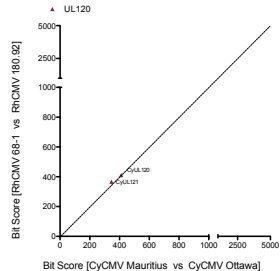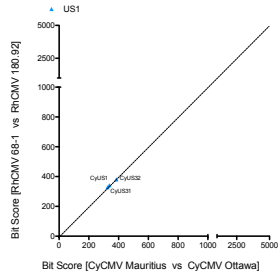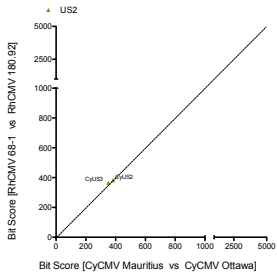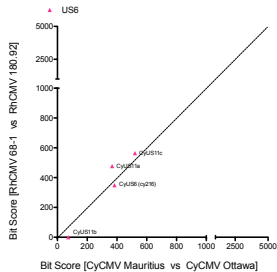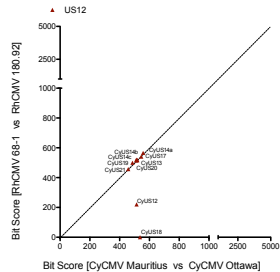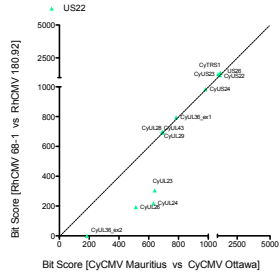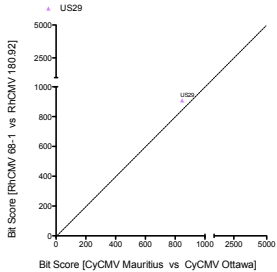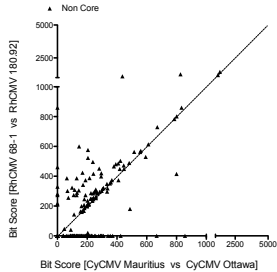

Supplement: Additional file 8: Figure S7. — Break down of two-dimensional bit-score plots between CMV Mauritius-CyCMV Ottawa versus RhCMV 68-1-RhCMV 180.92 by ORF family. Graphs represent comparisons of ORF homologue bit-scores across three strains simultaneously. ORFs are coloured according to ORF family. CyCMV Mauritius names for ORF labels. (PDF 57 kb) [file 12864_2016_2588_MOESM8_ESM.pdf]

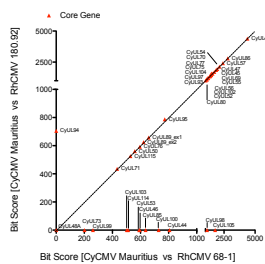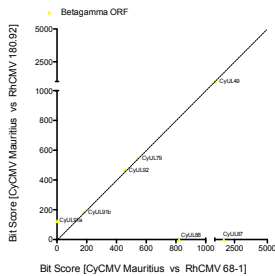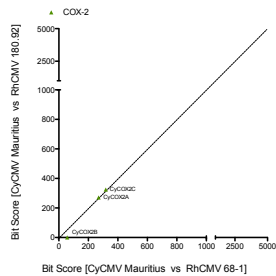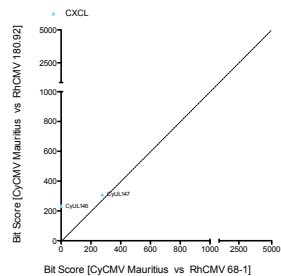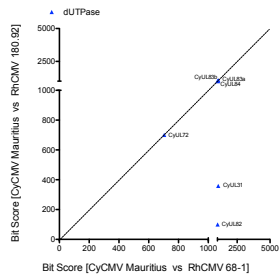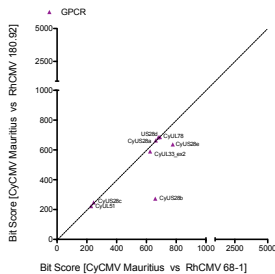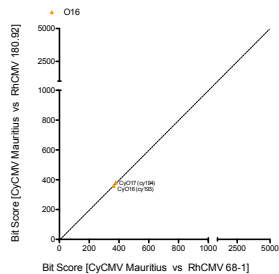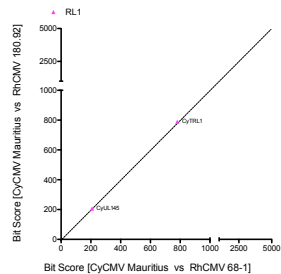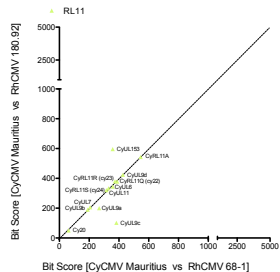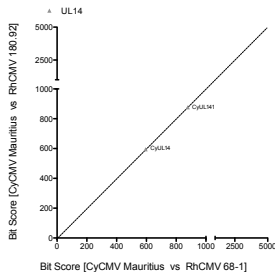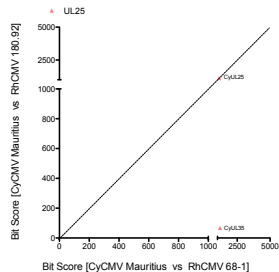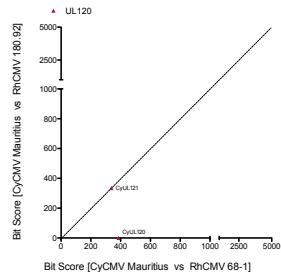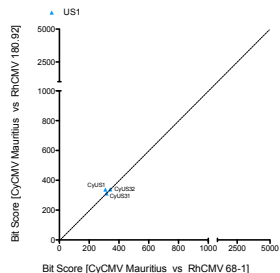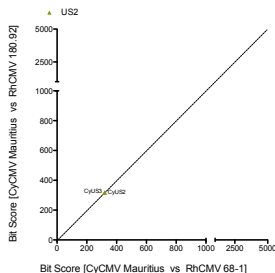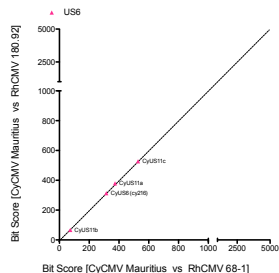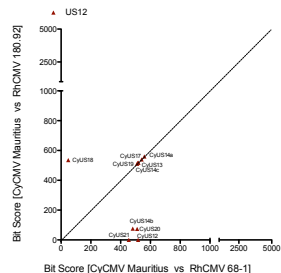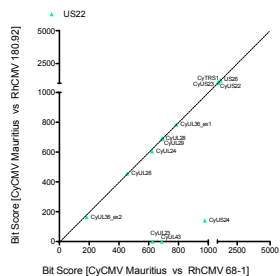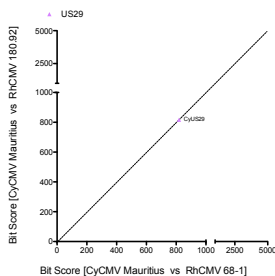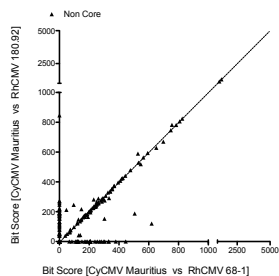

Supplement: Additional file 9: Figure S8. — Break down of two-dimensional bit-score plots between CyCMV Mauritius-RhCMV 68–1 versus CyCMV Mauritius-RhCMV 180.92 by ORF family. Graphs represent comparisons of ORF homologue bit-scores across three strains simultaneously. ORFs are coloured according to ORF family. CyCMV Mauritius names for ORF labels. (PDF 56 kb) [file 12864_2016_2588_MOESM9_ESM.pdf]

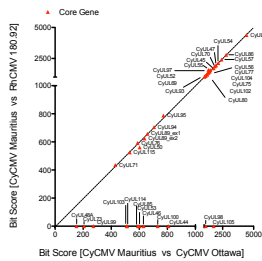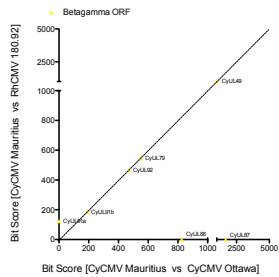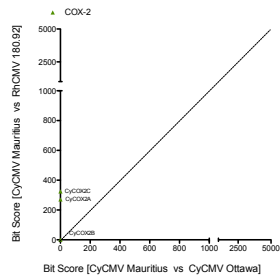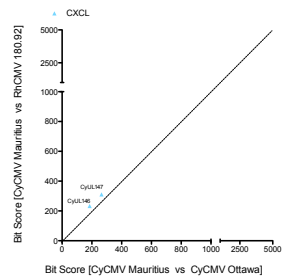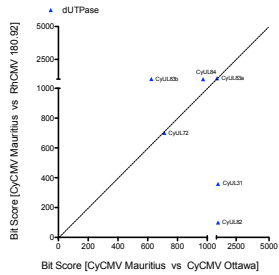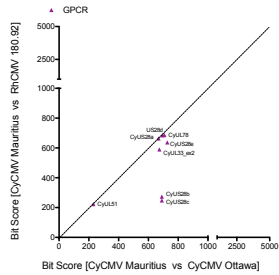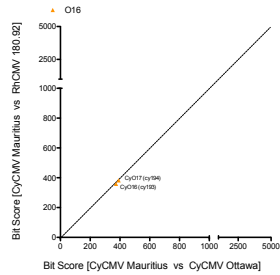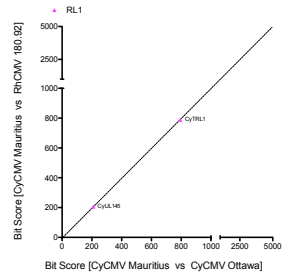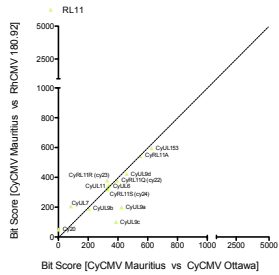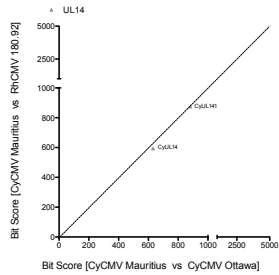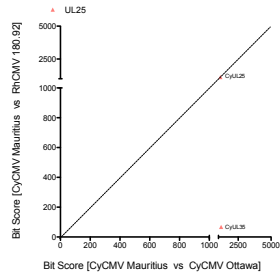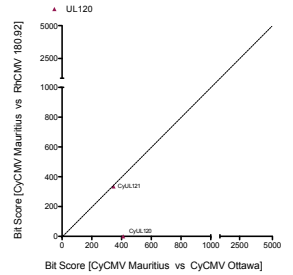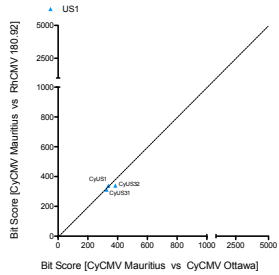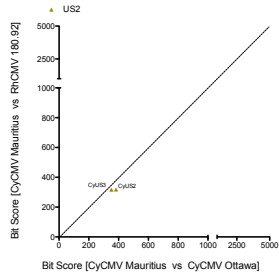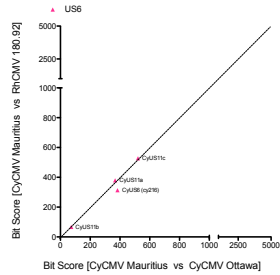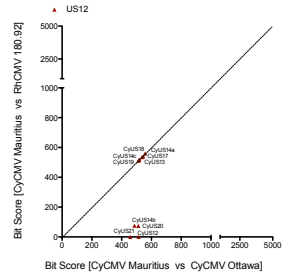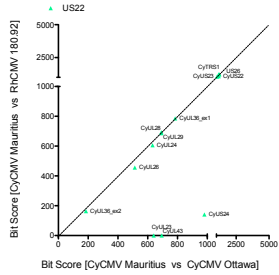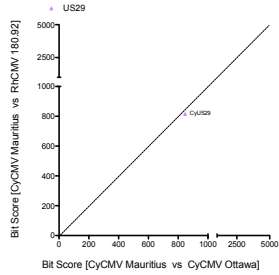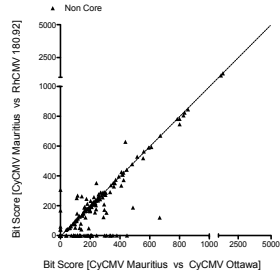

Supplement: Additional file 10: Figure S9. — Break down of two-dimensional bit-score plots between CMV Mauritius-CyCMV Ottawa versus CyCMV Mauritius-RhCMV 180.92 by ORF family. Graphs represent comparisons of ORF homologue bit-scores across three strains simultaneously. ORFs are coloured according to ORF family. CyCMV Mauritius names used for ORF labels. (PDF 57 kb) [file 12864_2016_2588_MOESM10_ESM.pdf]

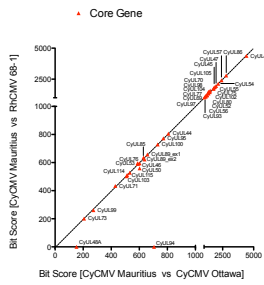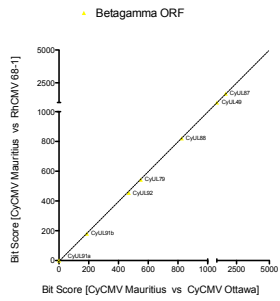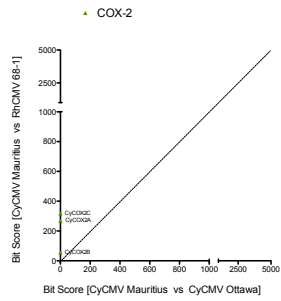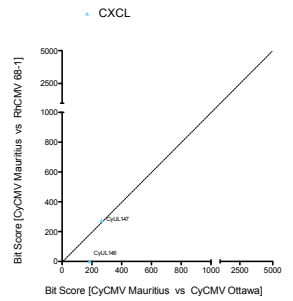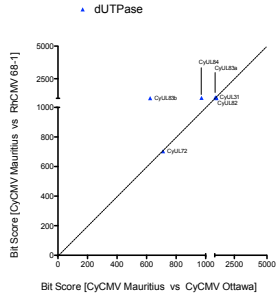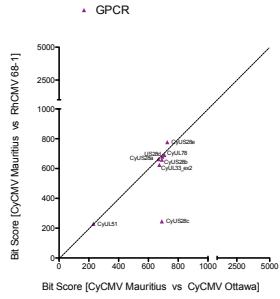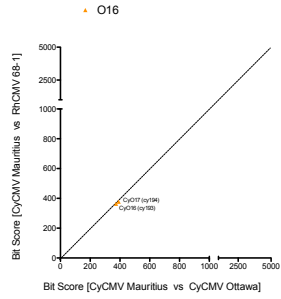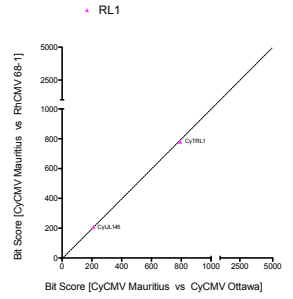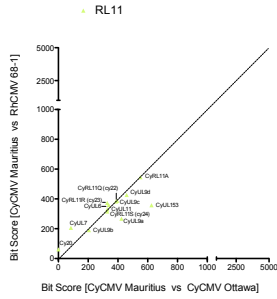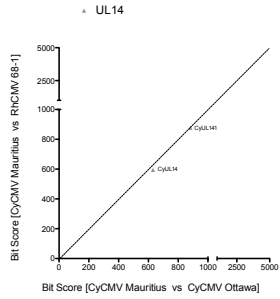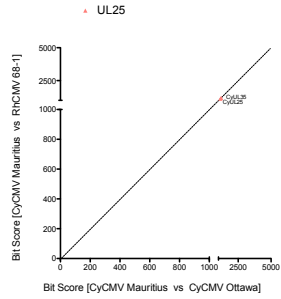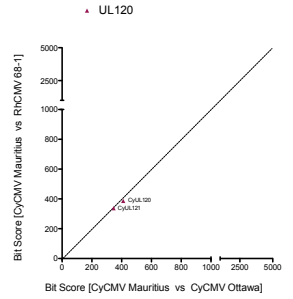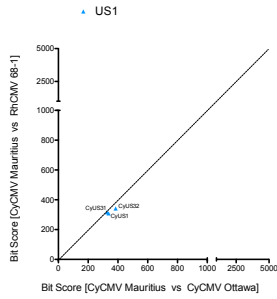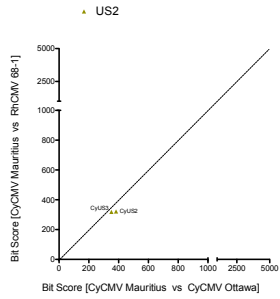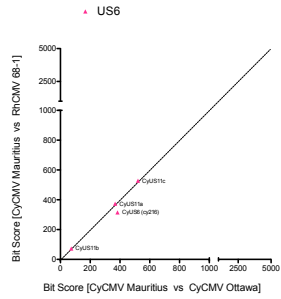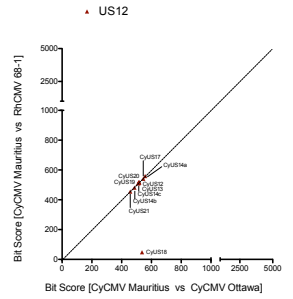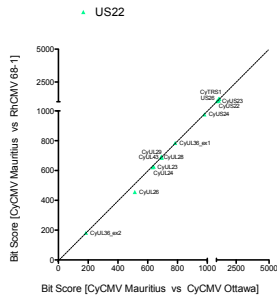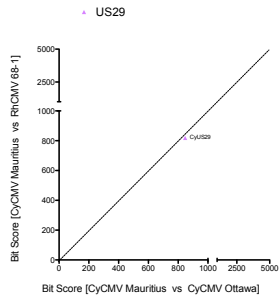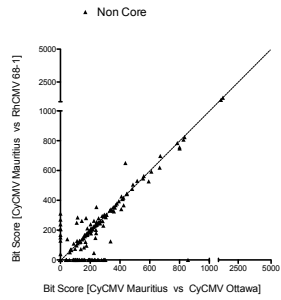

Supplement: Additional file 11: Figure S10. — Break down of two-dimensional bit-score plots between CMV Mauritius-RhCMV 68–1 versus CyCMV Mauritius-CyCMV Ottawa by ORF family. Graphs represent comparisons of ORF homologue bit-scores across three strains simultaneously. ORFs are coloured according to ORF family. CyCMV Mauritius names used for ORF labels. (PDF 57 kb) [file 12864_2016_2588_MOESM11_ESM.pdf]
