# Supplementary material for: A novel strain of cynomolgus macaque cytomegalovirus: implications for host-virus co-evolution
Source: BMC Genomics. 2016 Apr 5;17:277. doi: 10.1186/s12864-016-2588-3 (PMC4820910; doi:10.1186/s12864-016-2588-3)
Supplement: Additional file 2: Table S1. — ORFs of CyCMV Mauritius. Footnotes: 1 Functions annotated based on studies of HCMV [52] unless otherwise indicated. 2 Nearest homologous HCMV gene based on Bit score using BLASTP search. Strain of HCMV gene indicated with footnote. 3 Based on function of RhCMV68-1 homologue [19]. 4 Based on function of CyCMV Ottawa homologue [18]. 5 Based on studies of HCMV Towne-BAC [54]. 6 Inferred through function of homologous proteins [52]. 8 HCMV strain AD169. 9 HCMV strain AF1. 10 HCMV strain ASM72. 11 HCMV strain C154. 12 HCMV strain C425, 13 HCMV strain CINCY + Towne. 14 HCMV strain Coz. 15 HCMV strain David. 16 HCMV strain FRCMV-14L. 17 HCMV strain GSV6. 18 HCMV strain GSV9. 19 HCMV strain HAN1. 20 HCMV strain HAN13. 21 HCMV strain HAN16. 22 HCMV strain HAN20. 23 HCMV strain HAN3. 24 HCMV strain HAN38. 25 HCMV strain HKS40. 26 HCMV strain I-10. 27 HCMV strain IS17. 28 HCMV strain JHC. 29 HCMV strain JP. 30 HCMV strain L2. 31 HCMV strain Merlin. 32 HCMV strain RK (Human Herpesvirus 7). 33 HCMV strain TB40/E. 34 HCMV strain Toledo. 35 HCMV strain Towne. 36 HCMV strain TR. 37 HCMV strain U11. 38 HCMV strain U8. 39 HCMV strain VR1814. 40 HCMV strain 35. 41 HCMV strain 66. 42 HCMV strain 452. 43 HCMV strain 553. 44 HCMV strain 3157. 45 HCMV strain 3301. 46 HCMV strain 5234. 47 HCMV strain 401058. 48 HCMV strain 26M. 49 HCMV strain 51C. (PDF 158 kb) [file 12864_2016_2588_MOESM2_ESM.pdf]

ORF	Gene Family	Putative Function[1]	Translation		Strand	Size (aa)	Putative Molecular Mass (kDa)	Nearest HCMV homologue[2]	Non-Human Primate Homologues		
			Start	Stop					CyCMV Ottawa	RhCMV 68-1	RhCMV 180.92
CyTRL1	RL1	-	903	2675	+	590	65.808	RL01 $_{24}$	CyTRL1 (90.4%)	Rh01 (85.9%)	RhRL1 (86.7%)
Cy02	-	-	1589	1849	+	86	9.794	-	Cy02 (85.3%)	Rh02 (76.8%)	Rh2 (78%)
Cy03	-	-	1993	2730	+	245	26.341	-	Cy03 (69.4%)	-	Rh2.1 (71%)
Cy04	-	-	2727	3224	+	165	18.209	-	Cy04 (95.2%)	Rh03 (91.3%)	Rh3 (90.7%)
Cy05	-	-	3510	2812	-	232	27.064	-	Cy05 (97%)	Rh04 (90.9%)	Rh4 (90.5%)
CyO1 (Cy06)	-	-	2860	3162	+	100	11.511	-	Cy06 (98%)	-	Rh3.1 (94%)
Cy07	-	-	3197	3682	+	161	18.051	-	Cy07 (96.9%)	-	Rh3.2 (92%)
CyRL11A	RL11	IgG Fc binding glycoprotein modulator of antibody activity	3637	4458	+	273	30.151	-	CyRL11 (97.4%)	Rh05 (96%)	Rh5 (95.6%)
CyRL11B (Cy09)	-	-	4821	5303	+	160	18.479	-	Cy09 (44.2%)	Rh06 (86.4%)	Rh6 (86.2%)
CyRL11C (Cy10)	-	-	5415	6038	+	208	23.627	-	Cy10 (58.6%)	Rh07 (59.5%)	Rh7 (60%)
CyRL11D (Cy11)	-	-	6201	6812	+	203	23.135	-	Cy11 (31.2%)	Rh08 (33.8%)	Rh8 (29.1%)
CyO3	-	-	6821	7039	+	72	8.144	-	-	-	-
CyO7	-	-	7795	8019	+	74	8.419	-	-	-	-
CyCOX2A	COX-2	Putative COX-2 Homologue[6]	8563	8081	-	160	16.88	-	-	Rh10 (94.3%)	Rh10 (94.3%)
CyCOX2B	COX-2	Putative COX-2 Homologue[6]	8728	8579	-	49	6.126	-	-	Rh10 (92.6%)	-
CyCOX2C	COX-2	Putative COX-2 Homologue[6]	9357	8743	-	204	23.458	-	-	Rh10 (98%)	Rh10 (98.1%)
CyUL7	RL11	Putative membrane glycoprotein	9040	9480	+	146	16.945	-	CyUL7 (90%)	Rh11 (89.9%)	Rh11 (89.9%)
CyUL6	RL11	Putative membrane glycoprotein	9538	10128	+	192	21.782	UL6 $_{34}$	CyUL6 (82.2%)	Rh20 (88.8%)	RhUL6 (88.9%)
CyUL9a	RL11	Temperance factor & Putative membrane glycoprotein	10160	10843	+	227	26.516	UL9 $_{10}$	CyUL9 (97.4%)	Rh21 (61.8%)	Rh21 (66.7%)
Cy20	RL11	Putative membrane glycoprotein[6]	10997	11146	+	49	6.069	-	-	Rh22 (58.7%)	Rh22 (66.7%)
CyUL11	RL11	Membrane glycoprotein	11044	11721	+	225	25.308	UL9 $_{23}$	CyUL11 (85.4%)	Rh23 (79.7%)	RhUL1 (80.6%)
CyUL9b	RL11	Temperance factor & Putative membrane glycoprotein	11772	12134	+	120	13.631	UL9 $_{21}$	CyUL9 (95%)	Rh24 (88.3%)	Rh24 (88.3%)
CyUL9c	RL11	Temperance factor & Putative membrane glycoprotein	12213	12887	+	224	24.673	UL9 $_{26}$	CyUL9 (92%)	Rh25 (92%)	Rh26 (29.5%)
CyUL9d	RL11	Temperance factor & Putative membrane glycoprotein	12892	13710	+	272	31.067	UL9 $_{21}$	CyUL9 (89%)	Rh26 (81.4%)	Rh26 (81.8%)
CyRL11Q (Cy22)	RL11	-	13855	14463	+	202	22.99	-	Cy22 (93.6%)	Rh27 (93.6%)	Rh27 (90.1%)
CyRL11R (Cy23)	RL11	-	14465	15088	+	207	23.948	-	Cy23 (75.4%)	Rh28 (86%)	Rh28 (88.4%)
CyRL11S (Cy24)	RL11	Putative membrane glycoprotein[3]	15163	16422	+	419	45.885	-	Cy24 (70.5%)	Rh29 (69.1%)	Rh29 (70.7%)
Cy25	-	-	16821	16498	-	107	11.613	-	Cy25 (97.2%)	Rh30 (97.2%)	Rh30 (96.1%)
CyUL13	-	Putative Secreted protein	16538	17848	+	436	50.654	-	CyUL13 (94.7%)	Rh31 (94%)	Rh31 (94.3%)
Cy28	-	-	17203	16760	-	147	16.953	-	Cy28 (95.8%)	Rh32 (89.1%)	Rh32 (89.1%)
CyUL14	UL14	Putative membrane glycoprotein	18123	19034	+	303	35.334	UL14 $_{31}$	CyUL14 (99.7%)	Rh33 (98%)	RhUL1 (97%)
CyO8	-	-	19309	19599	+	96	11.399	-	-	-	-
CyO9	-	-	19683	19880	+	65	7.73	-	-	-	-
Cy29	-	-	20149	19886	-	87	9.952	-	-	Rh34 (95.4%)	Rh34 (95.4%)
CyUL17 (Cy30)	-	Putative 7 transmembrane protein	19946	20272	+	108	12.773	-	Cy30 (95.4%)	Rh35 (96.3%)	-
CyUL19	-	-	20598	20885	+	95	19.748	UL19 $_{22}$	CyUL19 (96.8%)	Rh35.1 (95.8%)	RhUL1 (94.7%)
CyUL20	-	T cell receptor gamma chain homologue	20994	22364	+	456	51.387	UL20 $_{13}$	CyUL20 (85.3%)	Rh36 (83.3%)	RhUL2 (83.8%)
CyUL21A	-	Fibroblast Temperance factor & CC chemokine-binding protein	22831	22468	-	120	13.841	UL21A $_{9}$	CyUL21A (99.2%)	Rh37 (98.3%)	RhUL2 (99.2%)
Cy34	-	-	23375	23187	-	62	6.33	-	Cy34 (68%)	-	-
Cy35	-	-	23529	23314	-	71	8.232	-	-	Rh39 (88.9%)	Rh39 (71.4%)
CyUL23	US22	Temperance factor & Tegument protein	24864	23926	-	312	35.906	UL23 $_{34}$	CyUL23 (98.1%)	Rh40 (95.8%)	-
Cy36	-	-	24746	24994	+	82	9.49	-	Cy36 (97.6%)	Rh41 (92.7%)	Rh41 (91.5%)
CyUL24	US22	Temperance factor & Tegument protein	25850	24921	-	309	35.228	UL24 $_{34}$	CyUL24 (100%)	Rh42 (98.1%)	UL24 (95.8%)
CyUL25	UL25	Temperance factor & Tegument phosphoprotein	25917	27683	+	588	67.28	UL25 $_{31}$	CyUL25 (98.3%)	Rh43 (95.1%)	RhUL25 (95.6%)
CyUL26	US22	Transcriptional activator of major immediate early promoter & regulator of tegument protein phosphorylation	28499	27744	-	251	28.188	UL26 $_{35}$	CyUL26 (98.8%)	Rh44 (97.5%)	RhUL26 (97.5%)
CyUL27	-	Maribavir resistance	30186	28453	-	577	65.694	pUL27 $_{43}$	CyUL27 (99.1%)	Rh46 (97.6%)	RhUL27 (97.6%)
CyUL28	US22	-	31284	30271	-	337	38.679	UL28 $_{8}$	CyUL28 (98.8%)	Putatative Rh50/47 (98.2%)	RhUL28 (98.5%)
Cy42	-	-	30496	30957	+	153	17.046	-	Cy42 (91.1%)	Rh48 (87.6%)	-
Cy43	-	-	31396	31794	+	132	15.247	-	Cy43 (96.3%)	Rh49 (91.7%)	Rh49 (92.4%)
CyUL29	US22	Temperance factor	32424	31414	-	336	38.843	UL29 $_{8}$	CyUL29 (99.4%)	Rh50 (98.5%)	UL29 (98.2%)
Cy45	-	-	31961	31545	-	138	15.642	-	Cy45 (97%)	Rh51 (93.3%)	Rh51 (94%)
Cy46	-	-	31810	32322	+	170	19.225	-	Cy46 (96.5%)	Rh52 (92.1%)	-
Cy47	-	-	32476	32709	+	77	9.002	-	Cy47 (100%)	Rh52 (100%)	-
CyUL30	-	-	32764	32498	-	88	10.32	UL30 $_{33}$	CyUL30 (100%)	Rh50.1 (96.4%)	-
Cy49	-	-	33188	32754	-	144	17.408	UL30A $_{8}$	Cy49 (97.7%)	Rh53 (94.8%)	Unknown (94.5%)
CyUL31	dUTPase	Immediate early protein	33071	34696	+	541	61.074	UL31 $_{35}$	CyUL31 (99.3%)	Rh54 (97.8%)	UL31 (94.7%)
CyUL32	-	Major Tegument phosphoprotein (pp150/ binds to capsids/ highly immunogenic	36839	34707	-	710	79.61	pp150 $_{35}$ (UL32)	CyUL32 (97.3%)	Rh55 (92.4%)	RhUL32 (92.5%)
Cy52_ex1	-	-	36793	36945	+	50	6.024	-	Cy52 exon 1 (100%)	-	G protein coupled UL33 like (94%)
CyUL33_ex2	GPCR	Constitutive signalling	37207	38196	+	329	37.331	-	CyUL33 (98.5%)	G protein coupled UL33 like (93%)	RhUL33 exon 2 (95.7%)
CyUL34	-	Repressor of US3 transcrition	38401	39258	+	285	32.858	UL34 $_{34}$	CyUL34 (99.6%)	Rh57 (96.6%)	RhUL34 (99.3%)
Cy54	-	-	38715	39101	+	128	13.906	-	Cy54 (93%)	Rh58 (91.4%)	Rh58 (93.8%)
CyUL35	UL25	UL82 interacting Tegument phosphoprotein & regulator of virion transactivation and assembly	39321	41093	+	590	66.924	UL35 $_{9}$	CyUL35 (99.3%)	Rh59 (98.1%)	RhUL25 (22%)
CyUL36_ex1	US22	Immediate early tegument protein / Inhibitor of caspase-8-induced apoptosis	42380	41217	-	387	44.731	UL36 $_{44}$	CyUL36 (97.4%)	Rh60 (97.4%)	RhUL36 (97.7%)
Cy57	-	-	41487	41786	+	99	11.289	-	Cy57 (93.9%)	-	Rh59.1 (89.9%)
CyUL36_ex2	US22	Immediate early tegument protein / Inhibitor of caspase-8-induced apoptosis	42712	42434	-	92	10.341	UL36 $_{34}$	CyUL36 (97.8%)	Rh61 (94.6%)	RhUL36 (97.4%)
CyUL37_ex1	-	Immediate early protein / Mitochondrial inhibitor of apoptosis	43628	42810	-	272	31.148	UL37 $_{40}$	CyUL37 (97.1%)	Rh62 (98.2%)	RhUL37 (98.2%)
Cy59	-	-	43830	43961	+	43	5.049	-	Cy59 (82.5%)	Rh63 (77.5%)	-
CyUL38	-	Virion Envelope Glycoprotein	44816	43932	-	294	33.344	UL38 $_{35}$	CyUL38 (99.3%)	Rh64 (96.6%)	RhUL38 (96.9%)
Cy61	-	-	44001	44366	+	121	13.733	-	Cy61 (95.9%)	Rh65 (94.2%)	Rh65 (93.4%)
Cy62	-	-	44562	44861	+	99	11.442	-	Cy62 (91.9%)	-	Rh65.1 (91.9%)
CyUL37_ex2	-	Immediate early protein / Mitochondrial inhibitor of apoptosis	45142	44858	-	94	10.703	UL37 $_{45}$	CyUL37 (94.8%)	Rh66 (93.6%)	RhUL37 (93.4%)
CyUL40 (Cy63)	-	-	46023	45493	-	176	18.952	-	Cy63 (100%)	Rh67 (93.2%)	-
CyUL41A	-	Virion Envelope Protein	46340	46101	-	79	9.408	UL41A $_{31}$	CyUL41A (100%)	Rh67.1 (97.5%)	RhUL41 (98.7%)
CyUL42	-	Putative membrane protein	46858	46472	-	128	14.102	UL42 $_{20}$	CyUL42 (91.4%)	Rh68 (92.3%)	RhUL42 (91.4%)
CyUL43	US22	Tegument protein / Putative multiple transmembrane ptotein	47843	46842	-	333	38.49	UL43 $_{31}$	CyUL43 (99.7%)	Rh69 (98.2%)	-
CyUL44	Core Gene	DNA polymerase accessory subunit / Increases DNA polymerase product length	49134	47962	-	390	44.011	UL44 $_{8}$	CyUL44 (99%)	Rh70 (98.5%)	-
Cy68	-	-	48364	48840	+	158	17.891	-	Cy68 (96.2%)	Rh71 (93.7%)	-
CyUL45	Core Gene	Enzymatically inactive large subunit ribonucleoride reductase homologue tegument protein	51927	49375	-	850	96.916	UL44 $_{33}$	CyUL45 (99.6%)	Rh72 (98.1%)	Rh72 (97.9%)
Cy70	-	-	49636	49827	+	63	6.807	-	Cy70 (98.4%)	Rh73 (92.1%)	-
Cy71	-	-	51413	51715	+	100	11.284	-	Cy (0%)	Rh74 (99%)	-
CyUL46	Core Gene	Component of Capsid triplexes (minor capsid binding protein)	52818	51946	-	290	33.128	UL46 $_{31}$	CyUL46 (99.7%)	Rh75 (98.3%)	-

Table continued on next page

ORF	Gene Family	Putative Function[1]	Translation		Strand	Size (aa)	Putative Molecular Mass (kDa)	Nearest HCMV homologue[2]	Non-Human Primate Homologues		
			Start	Stop					CyCMV Ottawa	RhCMV 68-1	RhCMV 180.92
CyUL47	Core Gene	UL48 binding tegument protein involved in intracellular capsid transport	52817	55693	+	958	110.632	UL37$_{28}$	CyUL47 (99.1%)	Rh76 (97.7%)	RhUL47 (97.6%)
CyUL48	Core Gene	Largest Capsid protein involved in intracelluar capsid transport transport	55714	62253	+	2179	246.998	UL48$_{24}$	CyUL48 (99.2%)	Rh78 (98.5%)	RhUL48 (98.6%)
Cy75	-	-	56213	56605	+	130	15.039	-	Cy75 (92.3%)	-	Rh78.1 (89.2%)
CyUL48A	Core Gene	Smallest Capsid protein (located on tip of hexons) putatively involved in capsid transport	62547	62326	-	73	8.439	UL48A$_{31}$	CyUL48a (100%)	-	-
Cy77	-	-	62405	63091	+	228	24.686	-	Cy77 (99.5%)	Rh79 (99.1%)	-
CyUL49	Betagamma ORF	Membrane protein	64009	62540	-	489	56.125	UL49$_{24}$	CyUL49 (99.6%)	Rh80 (99.2%)	RhUL49 (99.4%)
CyUL50	Core Gene	Inner nuclear membrane protein involved in nuclear egress of capsids	64880	63999	-	293	32.346	UL50$_{45}$	CyUL50 (100%)	Rh81 (99.3%)	RhUL50 (99.3%)
CyUL51	GPCR	Terminase component (TER3) / DNA packaging	65241	64906	-	111	12.356	UL33$_{8}$	CyUL51 (100%)	Rh82 (99.1%)	RhUL51 (97.3%)
CyUL52	Core Gene	Putatively involved in capsid transport in nucleus	65279	66937	+	552	62.559	UL52$_{33}$	CyUL52 (98.7%)	Rh83 (98.7%)	RhUL52 (98.9%)
Cy82	-	-	65697	65311	-	128	13.632	-	Cy82 (98.8%)	Rh84 (98.8%)	Rh84 (98.8%)
CyUL53	Core Gene	Tegument protein / Nuclear matrix protein /Capsid and nuclear egress lamina protein	66930	67796	+	288	33.024	UL53$_{35}$	CyUL53 (99.3%)	Rh85 (98.6%)	-
Cy83	-	-	67772	67341	-	143	16.017	-	-	Rh86 (97.9%)	-
CyUL54	Core Gene	DNA polymerase catalytic subunit (POL)	70881	67774	-	1035	116.571	UL54$_{32}$	CyUL54 (99.9%)	Rh87 (98.9%)	RhUL54 (98.8%)
Cy86	-	-	70702	71079	+	125	14.097	-	Cy86 (96%)	Rh88 (96.8%)	Rh88 (96%)
CyUL55	Core Gene	Virion Envelope Glycoprotein B (gB) / Forms homomultimers / Heparin binding / Role in entry and signalling	73449	70900	-	849	97.466	UL55$_{33}$	CyGlycoprotein B (cyUL55) (77.7%)	Rh89 (88.7%)	RhUL55 (88%)
Cy87	-	-	72895	72987	+	30	3.516	-	-	Rh90 (72.4%)	Rh90 (72.4%)
CyUL57	Core Gene	ssDNA binding protein	75721	73415	-	768	88.185	UL57$_{32}$	CyUL57 (99.8%)	Rh92 (99.7%)	RhUL57 (99.6%)
Cy89	-	-	75785	74418	-	455	48.398	-	Cy89 (98.9%)	-	Rh91.1 (97.4%)
Cy90	-	Bat Herpesvirus Homology[6]	75666	75842	+	58	6.832	-	-	-	-
CyUL56	Core Gene	Terminase component (TER2) / binds DNA packaging motif & nuclease activity	79359	75868	-	1163	129.202	UL56$_{26}$	CyUL56 (99.1%)	Rh91 (99%)	RhUL56 (99.1%)
Cy91	-	-	80825	80577	-	82	9.184	-	Cy91 (87.8%)	Rh93 (86.6%)	Rh93 (86.6%)
Cy92	-	-	80934	80674	-	86	8.889	-	Cy92 (71.4%)	-	-
Cy93	-	-	81372	81064	-	102	10.76	-	Cy93 (98.9%)	Rh95 (96.7%)	Rh95 (97.8%)
CyUL69	Core Gene	Multiple regulatory protein tegument protein / Contributes to cell cycle block and nucleocytoplasmic export of unspliced mRNA	85484	83157	-	775	87.204	UL69$_{8}$	CyUL69 (98.1%)	Rh97 (96.7%)	RhUL69 (96.9%)
Cy95	-	-	84148	84456	+	102	11.793	-	Cy95 (100%)	Rh98 (99%)	-
Cy96	-	-	84699	85481	+	260	28.354	-	Cy96 (96.9%)	Rh99 (93.8%)	Rh99 (94.2%)
Cy98	-	-	86693	86884	+	63	7.075	-	Cy98 (88.9%)		Rh99.1 (88.9%)
CyUL70	Core Gene	DNA Helicase primase subunit involved in DNA unwinding	88156	85418	-	912	105.521	Ul70$_{8}$	CyUL70 (99.6%)	Rh100 (98.5%)	RhUL70 (98.5%)
CyUL71	Core Gene	Tegument protein putatively involved in Cytoplasmic egress	88169	88885	+	238	26.247	UL51$_{20}$	CyUL71 (98.3%)	Rh100.1 (95%)	RhUL71 (95.4%)
CyUL72	dUTPase	Enzymatically inactive dUTPase homologue virion protein	89984	88953	-	343	39.208	UL72$_{37}$	CyUL72 (99.7%)	Rh101 (98.3%)	RhUL72 (98%)
CyUL73	Core Gene	Virion Envelope Glycoprotein N (gN) / Complexes with gM involved in entry	89979	90290	+	103	11.713	UL73	CyUL73 (97.1%)	Rh102 (96.2%)	None (0%)
CyUL74	Non-Core	Virion Envelope Glycoprotein O (gO) / Complexes with gH:gL involved in entry	91452	90271	-	393	46.01	UL74$_{11}$	CyUL74 (98.2%)	Rh103 (95.4%)	RhUL74 (95.2%)
CyUL74A	Non-Core	Virion Envelope Glycoprotein 24	91451	91621	+	56	6.415	UL74A$_{33}$	CyUL74A (100%)	-	-
CyUL75	Core Gene	Virion Envelope Glycoprotein H (gH) / Associates with gO & complexes with GL or UL128-131 involved in entry	93831	91669	-	720	81.442	UL75$_{45}$	CyUL75 (98.5%)	Rh104 (97.8%)	RhUL75 (97.6%)
CyUL76	Core Gene	Viral associated regulatory protein	93964	94848	+	294	32.85	UL24$_{8}$	CyUL76 (98.6%)	Rh105 (100%)	RhUL76 (99.7%)
CyUL77	Core Gene	DNA packaging & Portal Capping protein	94517	96304	+	595	67.331	UL25$_{31}$	CyUL77 (99.5%)	Rh106 (99.7%)	RhUL77 (99.5%)
Cy107	-	-	94996	94613	-	127	14.328	-	Cy107 (98.4%)	-	Rh106.1 (97.6%)
CyUL78	GPCR	Putative chemokine receptor protein	96431	97576	+	381	42.149	UL78$_{35}$	CyUL78 (92.9%)	Rh107 (91.3%)	RhUL78 (90.8%)
CyUL79	Betagamma ORF	-	98478	97678	-	266	30.455	UL79$_{24}$	CyUL79 (100%)	Rh108 (98.9%)	RhUL79 (99.2%)
CyUL80	Core Gene	Capsid maturation protease and capsid assemply scaffold protein precursor	98477	100315	+	612	66.39	Ul80$_{37}$	CyUL80 (98.7%)	Rh109 (98%)	RhUL80 (98%)
CyUL82	dUTPase / UL82	Tegument phosphoprotein (pp65) (lower matrix protein) / Virion transactivator / degrades	102080	100431	-	549	61.672	Ul82$_{36}$	CyUL82 (98.9%)	Rh110 (94.8%)	RhUL83 (24.8%)
CyUL83a	dUTPase / UL82	Major Tegument phosphoprotein (pp65) (upper matrix protein) / Interferon response supresssion	103832	102210	-	540	62.106	UL83$_{39}$	CyUL83 (98.5%)	Rh111 (97%)	RhUL83 (96.9%)
Cy113	-	-	102658	102410	-	82	9.098	-	Cy113 (95.2%)	-	-
CyUL83b	dUTPase / UL82	Major Tegument phosphoprotein (pp65) / Interferon response supresssion	105532	103901	-	543	61.741	UL83$_{39}$	CyUL83 (95.8%)	Rh112 (96.9%)	RhUL83 (97.2%)
Cy115	-	-	104705	105151	+	148	16.367	-	Cy115 (90.3%)	Rh113 (91.9%)	Rh113 (91.2%)
CyUL84	dUTPase/UL82	Nucleocytoplasmic shuttling and DNA replication organization/Binds IE2	107189	105651	-	512	57.359	UL84$_{13}$	CyUL84 (97.9%)	Rh114 (99.4%)	RhUL84 (99.2%)
Cy118	-	-	105712	106080	+	122	13.366	-	Cy118 (98%)	Rh115 (97.5%)	Rh115 (96.7%)
Cy119	-	-	106140	105781	-	119	13.13	-	Cy119 (91.6%)	-	Rh115.1 (92.4%)
Cy120	-	-	106931	107389	+	152	16.124	-	Cy120 (98%)	Rh116 (99.3%)	-
CyUL85	Core Gene	Capsid triplex subunit[2]	108030	107104	-	308	34.656	UL85$_{31}$	CyUL85 (99.4%)	Rh117 (100%)	-
CyUL86	Core Gene	Major capsid protein	112122	108091	-	1343	151.345	UL86$_{28}$	CyUL86 (99%)	Rh118 (99.4%)	RhUL86 (99.3%)
Cy123	-	-	108656	108970	+	104	11.804	-	Cy123 (99%)	Rh119 (97.1%)	-
Cy124	-	-	110317	109583	-	244	28.846	-	Cy124 (96.3%)	Rh120 (97.5%)	-
CyUL87	Betagamma ORF	-	112137	114692	+	851	96.581	UL87$_{35}$	CyUL87 (99%)	Rh122 (98.7%)	-
CyUL88	Betagamma ORF	Terminase Component putatively involved in Cytoplasmic egress	114704	115906	+	400	45.605	UL88$_{13}$	CyUL88 (99.3%)	Rh123 (98.3%)	-
CyUL89_ex1	Core Gene	Terminase Component (TER1) / ATPase subunit	116850	115903	-	315	35.829	Ul89$_{27}$	CyUL89 (100%)	UL89 DNA packaging Protein (100%)	RhUL89 (100%)
Cy128	-	-	116566	117135	+	189	20.879	-	Cy128 (96.3%)	Rh125 (93.2%)	Rh125 (92.7%)
CyUL91a	Betagamma ORF	Essential for transcription of true late (γ2) genes[5]	116849	117040	+	63	7.289	UL91$_{14}$	-	-	RhORF6 (100%)
CyUL91b	Betagamma ORF	-	117167	117475	+	102	10.84	UL91$_{35}$	CyUL91 (98.1%)	Rh126 (93.2%)	RhUL91 (96.1%)
CyUL92	Betagamma ORF	-	117363	118073	+	236	26.494	UL92$_{35}$	CyUL92 (98.3%)	Rh127 (97.5%)	RhUL92 (99.2%)
CyUL93	Core Gene	Tegument protein putatively involved in Capsid transport	118039	119604	+	521	59.752	UL17$_{29}$	CyUL93 (97.1%)	Rh128 (97.7%)	RhUL93 (97.7%)
Cy132	-	-	118200	118520	+	106	11.189	-	Cy132 (96.2%)	-	Rh128.1 (92.5%)
CyUL94	Core Gene	ssDNA binding cytoplasmic egress tegument protein	119480	120520	+	346	37.682	UL16$_{9}$	CyUL94 (98%)	-	RhUL94 (97.4%)
CyUL89_ex2	Core Gene	Terminase Component / ATPase subunit	121440	120517	-	307	35.795	Ul89$_{35}$	CyUL89 (99.3%)	UL89 (99.7%)	RhUL89 (100%)
CyUL95	Core Gene	Putative encapsulation chaparone protein	121439	122734	+	431	47.029	UL95$_{8}$	CyUL95 (99.2%)	Rh130 (99.2%)	RhUL95 (99%)
CyUL96	-	Tegument protein	122731	123120	+	129	14.796	UL14$_{31}$	CyUL96 (100%)	Rh131 (97.7%)	RhUL96 (96.9%)
CyUL97	Core Gene	Viral serine-threonine protein kinase tegument protein	123177	125003	+	608	67.997	UL97$_{30}$	CyUL97 (99.2%)	Rh132 (95.9%)	RhUL97 (96.4%)
Cy133	-	-	124136	123822	-	104	11.853	-	-	Rh133 (94.2%)	-
Cy134	-	Murid herpesvirus homology[6]	124487	124248	-	79	8.681	-	-	-	-
CyUL98	Core Gene	DNAse	125054	126724	+	556	63.381	UL98$_{33}$	CyUL98 (99.6%)	Rh134 (99.8%)	-
Cy138	-	-	125967	125689	-	92	10.188	-	Cy138 (100%)	Rh135 (98.9%)	-
Cy139	-	-	126454	126005	-	149	16.874	-	Cy139 (99.3%)	Rh136 (98.7%)	Rh136 (98%)
CyUL99	Core Gene	Myristylated tegument phosphoprotein (pp28) involved in cytoplasmic egress	126661	127116	+	151	16.654	UL99$_{33}$	CyUL99 (92.7%)	Rh137 (92.8%)	-

Table continued on next page

ORF	Gene Family	Putative Function[1]	Translation		Strand	Size (aa)	Putative Molecular Mass (kDa)	Nearest HCMV homologue[2]	Non-Human Primate Homologues		
			Start	Stop					CyCMV Ottawa	RhCMV 68-1	RhCMV 180.92
CyUL100	Core Gene	Virion Envelope Glycoprotein M (gM) / Complexes with gN involved in entry	128353	127283	-	356	41.054	UL100 $_{37}$	CyUL100 (99.2%)	Rh138 (98.6%)	-
CyUL102	Core Gene	DNA Helicase primase subunit	128542	130716	+	724	80.418	UL102 $_{36}$	CyUL102 (99%)	Rh139 (98.2%)	RhUL10 (98.1%)
CyUL103	Core Genes	Tegument protein putatively involved in nuclear egress /envelope fusion protein	131493	130738	-	251	28.842	UL7 $_{31}$	CyUL103 (98.4%)	Rh140 (97.2%)	-
CyUL104	Core Gene	Capsid Portal Protein[4]/DNA encapsulation	133390	131420	-	656	75.513	Ul104 $_{24}$	CyUL104 (99.8%)	Rh141 (99.2%)	RhUL104 (99.1%)
CyUL105	Core Gene	DNA Helicase primase subunit	133227	135806	+	859	97.481	UL105 $_{34}$	CyUL105 (99.4%)	Rh142 (99.5%)	-
Cy146	-	-	133376	133828	+	150	16.912	-	Cy146 (98%)	-	Rh142.1 (95%)
Cy147	-	-	136674	136805	+	43	5.151	-	Cy147 (100%)	-	Rh142.3 (97.6%)
Cy148_ex1	-	Latency-associated viral Interleukin 10[4]	139102	139299	+	65	7.05	-	Cy148_exon 1 (96.9%)	Interleukin-10-like protein precursor (93.8%)	RhUL111 (96.8%)
CyUL111.5A_ex2	-	Latency-associated viral Interleukin 10[4]	139187	139648	+	153	17.642	-	CyUL111.5A (96.1%)	Rh143 (93.5%)	RhUL111a (91%)
Cy148_ex3	-	Interleukin 10 like protein precursor[4]	140026	140115	+	29	3.312	-	Cy148_ex3 (100%)	Interleukin-10-like protein precursor (91.3%)	RhUL111a (91.3%)
CyUL112_ex1	-	Early Phosphoprotein (p50)	140520	141317	+	265	28.286	UL112 $_{24}$	CyUL112 (94.3%)	Rh144/Rh145 (94.9%)	RhUL112 (90.6%)
CyUL112/113_ex2	-	Early Phosphoprotein (p84)[4]/Transcriptional activation and DNA replication	141416	142300	+	294	30.422	UL112 $_{9}$	CyUL112/113 (98.3%)	Rh144/Rh145 (94.9%)	RhUL112 (94.9%)
CyUL114	Core Gene	Uracil-DNA glycosylase	143158	142415	-	247	28.253	UL114 $_{34}$	CyUL114 (99.6%)	Rh146 (99.2%)	-
CyUL115	Core Gene	Virion Envelope Glycoprotein L (gL)/Associated with GH and complexes with gO or US128-130-131 for entry	143897	143121	-	258	29.176	UL115 $_{42}$	CyUL115 (99.6%)	Rh147 (98.4%)	RhUL115 (98.1%)
CyUL116	-	Putative membrane glycoprotein	144987	143908	-	359	38.048	UL116 $_{33}$	CyUL116 (96.7%)	Rh148 (92%)	RhUL116 (83.6%)
Cy153	-	-	144512	145009	+	165	16.703	-	Cy153 (99%)	Rh147.1 (89.2%)	-
Cy154	-	-	145126	144641	-	161	20.015	-	Cy154 (97.5%)	Rh149 (93.5%)	Rh149 (91.9%)
CyUL117	-	-	146117	144969	-	382	42.552	UL117 $_{20}$	CyUL117 (100%)	Rh150 (99.7%)	RhUL117 (99.5%)
Cy156	-	-	145953	146162	+	69	7.751	-	Cy156 (96.9%)	Rh149.1 (96.9%)	-
CyUL119_ex1	-	IgG Fc binding glycoprotein modulator of antibody activity/ virion glycoprotein	146743	146144	-	199	23.478	UL119 $_{20}$	CyUL119 (93%)	Rh141 (92.5%)	RhUL119 (92.5%)
Cy157_ex2	-	-	147504	146788	-	238	24.188	-	Cy157_exon 2 (88.8%)	Rh152 (93.9%)	RhUL119 (78.7%)
Cy158	-	-	147296	146979	-	105	11.721	-	Cy158 (100%)	Rh153 (87.6%)	Rh153 (87.5%)
CyUL120	UL120	Putative membrane glycoprotein	148149	147553	-	198	22.577	UL120 $_{38}$	CyUL120 (100%)	Rh154 (92.4%)	-
CyUL121	UL120	Putative membrane glycoprotein	148699	148151	-	182	21.046	UL121 $_{15}$	CyUL121 (97.8%)	Rh155 (95.6%)	Rh155 (95.1%)
CyUL122	-	Immediate Early 2 Transactivator/DNA-binding repressor activity & cell cycle modulation	150452	148965	-	495	53.824	IL122 $_{46}$	CyUL122 (92%)	Rh156 (94.3%)	RhUL122 (92.3%)
CyUL123	-	Major immediate early 1 co-transactivator/ enhancer of activation by IE2 & indirect effect on transcription machinery	152140	150953	-	395	44.25	IL123 $_{41}$	CyUL123 (61.9%)	Rh156 (83.5%)	RhUL123 (83.8%)
Cy161_ex3	-	Immediate early protein[4]	152582	152298	-	94	10.526	-	Cy161_exon 3 (65.9%)	Rh156 (80.6%)	RhUL122 (80.6%)
Cy162	-	-	152697	152954	+	85	10.229	-	Cy162 (93.1%)	Rh156.1 (90.7%)	-
Cy163	-	-	152806	153255	+	149	15.47	-	Cy163 (91.4%)	Rh156.2 (94.7%)	Rh156.2 (95.3%)
Cy161_ex4	-	Immediate early protein[4]	153101	152991	-	36	4.201	-	Cy161_exon 4 (94.9%)	Rh156 (IE1) (87.2%)	RhUL123 (82.1%)
Cy165a	-	-	153246	153512	+	88	9.609	-	Cy165 (43.1%)	-	-
CyUL126	-	Putative Map Kinase interacting Kinase[6]	153942	153796	-	48	5.661	UL126 $_{8}$	CyUL126 (97.9%)	-	-
Cy166	-	-	154358	154507	+	49	5.827	-	Cy166 (98%)	-	Rh157.1 (59.1%)
Cy165b	-	-	154811	154374	-	145	16.433	-	Cy165 (93.8%)	-	Rh157.1 (90.3%)
Cy165c	-	-	154386	154520	+	44	4.918	-	Cy165 (57.9%)	-	Rh157.1 (55.3%)
Cy167	-	-	154492	154695	+	67	7.168	-	Cy167 (94%)	-	Rh157.3 (55.1%)
Cy170	-	-	155050	154517	-	177	20.109	-	Cy170 (73.6%)	Rh157 (75%)	Rh157.3 (74.9%)
Cy168	-	-	154563	154796	+	77	8.476	-	Cy168 (90.9%)	-	Rh157.1 (56.3%)
Cy171	-	-	154735	155139	+	134	15.833	-	Cy171 (77.3%)	Rh157 (67.5%)	Rh157 (73.7%)
Cy165	-	-	154823	154927	+	34	3.748	-	Cy165 (66.7%)	-	
Cy172	-	-	154839	155084	+	81	9.239	-	Cy172 (75%)	-	Rh157.2 (69.3%)
CyUL128_ex3	-	Putative secreted CC chemokine/ Involved in endothelial and epithelial tropism/ complexes with gH & gL	156220	155894	-	108	12.075	-	CyUL128 (100%)	-	RhUL128 (97.2%)
CyUL128_ex2	-	Putative secreted CC chemokine/ Involved in endothelial and epithelial tropism/ complexes with gH & gL	156519	156430	-	29	3.372	-	-	-	RhUL128 (93.1%)
CyUL128_ex1	-	Putative secreted CC chemokine/ Involved in endothelial and epithelial tropism/ complexes with gH & gL	156798	156634	-	54	6.287	UL128 $_{17}$	CyUL128 (94.4%)	-	RhUL128 (93.6%)
Cy174	-	-	157495	156800	-	231	25.044	-	Cy174 (86.4%)	-	Rh157.4 (77%)
CyUL130	-	Putative Secreted protein involved in endothelial and epithelial tropism/ Complexes with gH & gL	157909	157616	-	97	11.524	UL130 $_{49}$	CyUL130 (95.9%)	-	RhUL130 (85.6%)
CyUL131A	-	Putative Secreted protein involved in endothelial and epithelial tropism/ Complexes with gH & gL	158506	158249	-	85	9.717	UL131A $_{18}$	CyUL131A (100%)	Rh157.6 (96.5%)	RhUL131a (96.5%)
CyUL132	-	Envelope Glycoprotein /post-entry temperance factor in fibroblasts	159215	158550	-	221	24.275	UL132 $_{38}$	CyUL132 (96.6%)	Rh160 (97.4%)	RhUL132 (96.6%)
CyUL148	-	Putative membrane glycoprotein	160261	159281	-	326	36.884	UL148 $_{49}$	CyUL148 (96.9%)	Rh159 (90.2%)	RhUL148 (87.5%)
CyUL147A	-	Putative membrane protein	160469	160272	-	65	6.957	-	-	-	-
CyUL147	CXCL	CXCL5 chemokine like secreted glycoprotein[6]	160927	160466	-	153	17.559	UL147 $_{47}$	CyUL147 (89.6%)	UL147 (96.7%)	UL147 (98.7%)
CyUL146	CXCL	CXCL5 chemokine like secreted glycoprotein[6]	161322	160978	-	114	12.922	-	CyUL146 (77.9%)	-	RhUL146 (98.2%)
Cy181	-	Putative CXC chemokine like protein[4]	161762	161568	-	64	7.234	-	Cy181 (90.6%)	-	RhUL146a (98.4%)
Cy182	-	Putative CXC chemokine like protein[4]	162211	161885	-	108	12.033	-	Cy182 (93.5%)	-	RhUL146b (95.4%)
Cy183	-	Putative CXC chemokine like protein[4]	162666	162334	-	110	12.498	-	Cy183 (99.1%)	Rh161 (35.2%)	Rh161.1 (93.6%)
Cy184	-	Putative CXC chemokine like protein[4]	163165	162749	-	138	15.767	-	Cy184 (97.8%)	Rh161 (97.3%)	Rh161.2 (97.1%)
CyUL145	RL1	-	163656	163351	-	101	11.259	UL145 $_{12}$	CyUL145 (100%)	Rh162 (99%)	RhUL145 (98%)
CyUL144	-	TNF receptor homologue membrane protein	164590	164075	-	171	18.686	-	CyUL144 (98.8%)	Rh163 (98.8%)	RhUL144 (98.8%)
CyUL141	UL14	NK cell inhibiting membrane glycoprotein	166101	164806	-	431	48.822	UL141 $_{34}$	CyUL141 (97.2%)	Rh164 (97%)	RhUL141 (96.8%)
CyO11	-	-	166412	166185	-	75	8.539	-	-	Rh164.1 (100%)	-
Cy188	-	-	167041	166595	-	148	17.239	-	Cy188 (98%)	Rh165 (94.6%)	Rh165 (94.6%)
CyO13 (Cy189)	-	-	167615	167088	-	175	19.479	-	Cy189 (93.1%)	Rh166 (94.9%)	Rh166 (94.3%)
CyO14 (Cy190)	-	-	168249	167746	-	167	18.177	-	Cy190 (97.6%)	Rh167 (96.4%)	RhUL148 (83.7%)
CyO15 (Cy191)	-	-	169162	168503	-	219	24651	-	Cy191 (97.3%)	Rh168 (95.9%)	Rh168 (95.4%)
Cy192	-	-	169259	169690	+	143	16.256	-	Cy192 (86.7%)	-	Rh168.1 (83.9%)
CyO16 (Cy193)	O16	-	169828	169265	-	187	20.667	-	Cy193 (94.7%)	Rh169 (92.5%)	Rh169 (91.4%)
CyO17 (Cy194)	O16	-	170528	169962	-	188	21.24	-	Cy194 (99.5%)	Rh170 (96.3%)	Rh170 (96.8%)
CyO18 (Cy195)	-	-	171370	170531	-	279	30.633	-	Cy195 (96.1%)	Rh171 (92.5%)	Rh171 (92.1%)
CyO19 (Cy196)	-	-	172101	171571	-	176	19.975	-	Cy196 (98.3%)	Rh172 (95.5%)	Rh172 (96.6%)
Cy197	-	-	171826	171978	+	50	5.352	-	Cy197 (96%)	-	Rh171.1 (92%)
CyUL153	RL11	Putative membrane protein[4]	173280	172153	-	375	40.855	RL13 $_{19}$	CyUL153 (96.5%)	Rh173 (57.9%)	Rh173 (90.9%)
CyO20 (Cy199)	-	-	175499	174417	-	360	40.025	-	Cy199 (92.5%)	Rh174 (93.6%)	Rh174 (93.6%)
Cy200	-	-	176196	176651	+	151	16.309	-	Cy200 (96.7%)	Rh175 (94%)	Rh175 (94.7%)
CyO21 (Cy201)	-	-	176992	176345	-	215	23.507	-	Cy201 (93.1%)	Rh176 (93%)	Rh176 (94.9%)

Table continued on next page

ORF	Gene Family	Putative Function[1]	Translation		Strand	Size (aa)	Putative Molecular Mass (kDa)	Nearest HCMV homologue[2]	Non-Human Primate Homologues		
			Start	Stop					CyCMV Ottawa	RhCMV 68-1	RhCMV 180.92
Cy202	-	-	177425	176958	-	155	17.491	-	Cy202 (93%)	Rh177 (79.9%)	Rh177 (79.2%)
CyO22 (Cy203)	-	-	177819	177052	-	255	28.114	-	Cy203 (91.8%)	Rh178 (87.5%)	Rh178 (86.4%)
Cy204	-	-	178111	177896	-	71	7.586	-	Cy204 (87.7%)	-	Rh178.2 (88.9%)
Cy205	-	-	178044	178142	+	32	3.415	-	Cy205 (87.9%)	-	Rh178.1 (76.7%)
Cy206	-	-	178231	178734	+	167	17.956	-	Cy206 (91.6%)	-	Rh178.3 (70.8%)
Cy207	-	-	178371	178694	+	107	11.841	-	Cy207 (87.9%)	-	Rh178.3 (84.6%)
Cy209	-	-	179424	178903	-	173	17.196	-	Cy209 (92.6%)	Rh180 (91.3%)	Rh180 (94.2%)
O23 (Cy208)	-	-	178994	179509	+	171	18.631	-	Cy208 (91.8%)	Rh179 (93%)	Rh179 (94.2%)
CyUS1	US1	-	180039	179533	-	168	19.267	US1 $_{37}$	CyUS1 (100%)	Rh181 (97.6%)	RhUS1 (98.8%)
Cy211	-	-	179888	180121	+	77	8.31	-	Cy211 (97.4%)		Rh180.1 (96.1%)
CyUS2	US2	Membrane glycoprotein/MHC processing and transport inhibition	180864	180274	-	196	23.139	US2 $_{9}$	CyUS2 (92.9%)	Rh182 (77.9%)	Rh182 (76.9%)
CyUS5 (Cy214)	-	-	181370	181735	+	121	13.682	-	Cy214 (89.7%)	Rh183 (86%)	Rh183 (86.8%)
CyUS3	US2	Immediate early membrane glycoprotein/MHC processing and transport inhibition	182062	181397	-	221	25.397	-	CyUS3 (94%)	Rh184 (92.6%)	Rh184 (92.6%)
Cy215	-	-	182860	182561	-	99	11.146	-	Cy215 (97%)	-	Rh184.1 (94.9%)
CyUS6 (Cy216)	US6	Putatively inhibits TAP peptide translocation	183514	182942	-	190	21.417	-	Cy216 (96.8%)	Rh185 (95.9%)	Rh185 (95.3%)
Cy217	-	-	184413	183724	-	229	27.241	-	Cy217 (83.1%)	Rh186 (83.2%)	Rh186 (84.1%)
CyUS11a	US6	MHC I degrading membrane glycoprotein	185230	184658	-	190	21.79	-	CyUS11 (92.6%)	Rh187 (94.7%)	Rh187 (94.7%)
CyUS11b	US6	MHC I degrading membrane glycoprotein	185343	185113	-	76	8.399	-	CyUS11 (86.8%)	Rh187 (86.8%)	Rh187 (78.9%)
Cy219	-	-	185813	185439	-	124	14.579	-	Cy219 (92.7%)	Rh188 (98.4%)	-
CyUS11c	US6	MHC I degrading membrane glycoprotein	187041	186100	-	313	36.658	US11 $_{34}$	CyUS11 (91.8%)	Rh189 (90.8%)	RhUS11 (90.8%)
Cy223	-	-	187938	187156	-	260	30.051	-	Cy223 (93.6%)	Rh191 (85.5%)	-
CyUS12	US12	Putative multiple transmembrane protein	187855	187313	-	180	19.783	US12 $_{34}$	CyUS12 (97.3%)	Rh190 (98.1%)	-
CyUS13	US12	Putative multiple transmembrane protein	188760	187996	-	254	29.726	US13 $_{36}$	CyUS13 (99.6%)	Rh192 (100%)	RhUS13 (99.6%)
Cy224	-	-	189251	188766	-	161	18.461	-	-	Rh193 (93.2%)	Rh193 (92.5%)
CyUS14a	US12	Putative multiple transmembrane protein	189706	188873	-	277	31.395	US14 $_{24}$	CyUS14 (98.2%)	Rh194 (98.6%)	Rh194 (98.9%)
CyUS14b	US12	Putative multiple transmembrane protein	190565	189837	-	242	27.324	US14 $_{31}$	CyUS14 (99.2%)	Rh195 (97.9%)	RhUS14 (26.8%)
CyUS14c	US12	Putative multiple transmembrane protein	191416	190658	-	252	29.503	US14 $_{9}$	CyUS14 (99.6%)	Rh196 (98.8%)	RhUS14 (99.2%)
Cy228	-	-	192247	191522	-	241	27.85	-	Cy228 (96.3%)	Rh197 (95.9%)	Rh197 (95.9%)
Cy229	-	-	192116	191802	-	104	12.443	-	Cy229 (92.1%)	-	Rh196.1 (91.3%)
CyUS17	US12	Putative multiple transmembrane protein	193049	192225	-	274	30.415	US17 $_{45}$	CyUS17 (98.5%)	Rh198 (97.8%)	RhUS17 (97.8%)
CyUS18	US12	Putative multiple transmembrane protein	193955	193155	-	266	30.015	US18 $_{31}$	CyUS18 (98.9%)	Rh192 (23.9%)	RhUS18 (98.5%)
CyUS19	US12	Temperance factor and putative multiple transmembrane protein	194860	194075	-	261	30.0734	US19 $_{24}$	CyUS19 (96.2%)	Rh200 (96.2%)	Rh200 (95.8%)
CyUS20	US12	Putative multiple transmembrane protein	195682	194921	-	253	28.578	US20 $_{34}$	CyUS20 (99.2%)	Rh201 (99.2%)	RhUS13 (27%)
CyUS21	US12	Membrane protein[6]	196416	195730	-	228	26.086	US21 $_{28}$	CyUS21 (100%)	Rh202 (98.2%)	-
CyUS22	US22	Tegument protein	198262	196538	-	574	65.879	US22 $_{9}$	CyUS22 (98.4%)	Rh203 (97.6%)	RhUS22 (97.4%)
CyUS23	US22	Tegument protein	200292	198421	-	623	72.542	US23 $_{34}$	CyUS23 (97.6%)	Rh204 (95.4%)	RhUS23 (95.7%)
Cy237	-	-	199133	198720	-	137	16.402	-	Cy237 (81.2%)	Rh206 (89%)	Rh206 (89.8%)
Cy238	-	-	198925	199248	+	107	12.684	-	Cy238 (94.2%)	Rh205 (88.4%)	Rh205 (88.4%)
Cy239	-	-	200077	200394	+	105	11.668	-	Cy239 (95.2%)	Rh208 (90.5%)	Rh208 (91.4%)
CyUS24	US22	Tegument protein	201746	200316	-	476	56.539	US24 $_{32}$	CyUS24 (99.6%)	Rh209 (99.2%)	RhUS23 (27.1%)
Cy241	-	-	201153	201338	+	61	6.852	-	Cy241 (91.7%)	Rh210 (85.2%)	-
US26	US22	-	203902	202109	-	597	67.842	US26 $_{9}$	CyUS26 (98.2%)	Rh211 (99.2%)	RhUS26 (99%)
Cy243	-	-	202878	202564	-	104	11.597	-	Cy243 (96.2%)	Rh212 (96.2%)	-
Cy244	-	-	202655	203170	+	171	19.018	-	Cy244 (95.3%)	Rh213 (94.7%)	-
CyUS28a	GPCR	CC and CXC chemokine receptor like virion envelope glycoprotein	204074	205060	+	328	37.497	US28 $_{42}$	CyUS28 (99.4%)	Rh214 (98.5%)	Rh214 (98.8%)
CyUS28b	GPCR	CC and CXC chemokine receptor like virion envelope glycoprotein	205395	206408	+	337	38.662	US28 $_{16}$	CyUS28 (99.1%)	Rh215 (93.8%)	Rh218 (38.1%)
CyUS28c	GPCR	CC and CXC chemokine receptor like virion envelope glycoprotein	206543	207544	+	333	38.093	US28 $_{36}$	CyUS28 (99.4%)	Rh218 (37.2%)	Rh218 (37.5%)
Cy248	-	-	207720	207439	-	93	10.528	-	Cy248 (96.7%)	Rh217 (89.1%)	Rh217 (91.3%)
US28d	GPCR	Membrane protein	207617	208639	+	340	39.229	US28 $_{48}$	CyUS28 (98.2%)	Rh218 (96.5%)	Rh218 (97.4%)
Cy250	-	-	208714	208409	-	101	11.545	-	Cy250 (96%)	Rh219 (94.1%)	Rh219 (93.1%)
CyUS28e	GPCR	Membrane protein	208784	210235	+	483	53.429	US28 $_{9}$	CyUS28 (85.6%)	Rh220 (98.6%)	RhUS28 (98.7%)
CyUS29	US29	Putative membrane glycoprotein	210396	211715	+	439	49.253	US29 $_{9}$	CyUS29 (97.3%)	Rh221 (94.8%)	RhUS29 (94.6%)
Cy253	-	-	210862	211185	+	107	12.663	-	Cy253 (97.2%)	Rh222 (95.3%)	-
CyUS30	-	Putative membrane glycoprotein	211633	212454	+	273	30.767	US30 $_{9}$	CyUS30 (98.2%)	Rh223 (96%)	Rh223 (96%)
Cy255	-	-	212933	212316	-	205	22.469	-	Cy255 (96.7%)	Rh224 (94.8%)	Rh224 (94.3%)
CyUS31	US1	-	212530	213015	+	161	18.547	US31 $_{8}$	CyUS31 (97.5%)	Rh225 (93.2%)	RhUS31 (91.9%)
CyUS32	US1	-	213142	213702	+	186	22.205	US32 $_{28}$	CyUS32 (100%)	Rh226 (96.8%)	RhUS32 (96.2%)
Cy259	-	-	213616	213422	-	64	7.203	-	Cy259 (100%)	Rh227 (93.7%)	-
CyO24 (Cy260)	-	-	213845	214150	+	101	10.809	-	Cy260 (98%)	Rh228 (93.1%)	Rh228 (88.3%)
CyO25	-	Putative Secreted protein	214774	214394	-	126	14.167	-	-	Rh228.1 (89.7%)	-
Cy261	-	Putative membrane protein	215251	214793	-	152	16.754	-	Cy261 (92.1%)	Rh229 (85.5%)	Rh229 (84.9%)
CyTRS1	US22	Immediate Early Tegument protein	216947	214872	-	691	77.289	TRS1 $_{35}$	CyTRS1 (96.7%)	Rh230 (92.5%)	RhTRS1 (92.8%)
